# Exosome-Based Molecular Transfer Activity of Macrophage-Like Cells Involves Viability of Oral Carcinoma Cells: Size Exclusion Chromatography and Concentration Filter Method

**DOI:** 10.3390/cells10061328

**Published:** 2021-05-27

**Authors:** Yanyin Lu, Takanori Eguchi, Chiharu Sogawa, Eman A. Taha, Manh Tien Tran, Toshiki Nara, Penggong Wei, Shiro Fukuoka, Takuya Miyawaki, Kuniaki Okamoto

**Affiliations:** 1 Department of Dental Pharmacology, Okayama University Graduate School of Medicine, Dentistry and Pharmaceutical Sciences, Okayama 700-8525, Japan; riku21@s.okayama-u.ac.jp (Y.L.); c.sogawa.3b@cc.it-hiroshima.ac.jp (C.S.); eman.taha@cira.kyoto-u.ac.jp (E.A.T.); trantienmanh1508@gmail.com (M.T.T.); 13898129859@163.com (P.W.); pkbs3srr@okayama-u.ac.jp (S.F.); k-oka@okayama-u.ac.jp (K.O.); 2 Department of Dental Anesthesiology and Special Care Dentistry, Okayama University Graduate School of Medicine, Dentistry and Pharmaceutical Sciences, Okayama 700-8525, Japan; miyawaki@md.okayama-u.ac.jp; 3 Advanced Research Center for Oral and Craniofacial Sciences, Okayama University Graduate School of Medicine, Dentistry and Pharmaceutical Sciences, Okayama 700-8525, Japan; 4 Department of Clinical Engineering, Faculty of Life Sciences, Hiroshima Institute of Technology, Hiroshima 731-5193, Japan; 5 Department of Biochemistry, Ain Shams University Faculty of Science, Cairo 11566, Egypt; 6 Center for iPS Cell Research and Application (CiRA), Kyoto University, Kyoto 606-8502, Japan; 7 Research Program for Undergraduate Students, Okayama University Dental School, Okayama 700-8525, Japan; pps64qnw@s.okayama-u.ac.jp; 8 O-NECUS Program of Okayama University Dental School, Department of Endodontics, School of Stomatology, China Medical University, Shenyang 110002, China; 9 Department of Orthopaedic Surgery, Okayama University Graduate School of Medicine, Dentistry and Pharmaceutical Sciences, Okayama 700-8558, Japan

**Keywords:** macrophage, exosomes, extracellular vesicles, molecular transfer, size exclusion chromatography and concentration filter (SEC-CF) method, heat shock proteins, oral carcinoma

## Abstract

Extracellular vesicles (EV) heterogeneity is a crucial issue in biology and medicine. In addition, tumor-associated macrophages are key components in cancer microenvironment and immunology. We developed a combination method of size exclusion chromatography and concentration filters (SEC-CF) and aimed to characterize different EV types by their size, cargo types, and functions. A human monocytic leukemia cell line THP-1 was differentiated to CD14-positive macrophage-like cells by stimulation with PMA (phorbol 12-myristate 13-acetate) but not M1 or M2 types. Using the SEC-CF method, the following five EV types were fractionated from the culture supernatant of macrophage-like cells: (i) rare large EVs (500–3000 nm) reminiscent of apoptosomes, (ii) EVs (100–500 nm) reminiscent of microvesicles (or microparticles), (iii) EVs (80–300 nm) containing CD9-positive large exosomes (EXO-L), (iv) EVs (20–200 nm) containing unidentified vesicles/particles, and (v) EVs (10–70 nm) containing CD63/HSP90-positive small exosomes (EXO-S) and particles. For a molecular transfer assay, we developed a THP-1-based stable cell line producing a GFP-fused palmitoylation signal (palmGFP) associated with the membrane. The THP1/palmGFP cells were differentiated into macrophages producing palmGFP-contained EVs. The macrophage/palmGFP-secreted EXO-S and EXO-L efficiently transferred the palmGFP to receiver human oral carcinoma cells (HSC-3/palmTomato), as compared to other EV types. In addition, the macrophage-secreted EXO-S and EXO-L significantly reduced the cell viability (ATP content) in oral carcinoma cells. Taken together, the SEC-CF method is useful for the purification of large and small exosomes with higher molecular transfer activities, enabling efficient molecular delivery to target cells.

## 1. Introduction

Extracellular vesicles (EVs) are small particles surrounded by lipid membrane that are released by cells of diverse organisms, including eukaryotes and prokaryotic cells. In pluricellular organisms, EVs exist in different body fluids such as blood, urine, saliva, bile, ascites, amniotic fluid, breast milk, pleural ascites, synovial fluid, and cerebral spinal fluid [1]. EVs contain various molecular cargo types, including proteins, nucleic acids, lipids, minerals, and metabolites [1,2,3]. Earlier studies classified EVs into the following three types: (1) exosomes (50–200 nm) originated from the endosome; (2) ectosomes, also called microvesicles (MVs) or microparticles (MPs) (100–1000 nm), generated by the budding and shedding of the plasma membrane of cells; and (3) apoptotic bodies (1–5 μm), also known as apoptosome, originated via blebbing of the plasma membrane [4,5,6]. Recent studies reported additional types of vesicles, such as oncosomes (100–400 nm), large oncosomes (1–10 μm) [7,8], matrix vesicles [9,10], migrasomes [11] (50 nm–3 μm), exopheres (~4 μm), and bacterial outer membrane vesicles (OMV) [12,13,14]. Additionally, non-membranous nanoparticles termed exomeres (~35 nm) involve metabolic enzymes and microtubule, hypoxia, coagulation proteins, glycosylation, and mTOR signaling [15].

EVs are also classified by their size, for example, large EVs (L-EVs, e.g., 200–2000 nm) and small EVs (S-EVs; e.g., 50–200 nm) depending on the context of the works [3,16] S-EVs often contain exosomes, while L-EVs could contain apoptotic bodies. Moreover, a previous study classified exosomes into larger exosomes (EXO-L, 90–120 nm) and smaller exosomes (EXO-S 60–80 nm) [15]. Exosomes usually contain tetraspanins such as CD9 and CD63, used as protein biomarkers to demonstrate the exosomes’ existence. Heat shock proteins (HSPs) are the regular components of EVs, while vesicle-free, extracellular HSPs have also been found [17,18,19,20].

Many methods have been developed for EV preparation, including ultracentrifugation (UC) method [21], filter centrifugation (FC), size exclusion chromatography (SEC) [22,23], affinity purification (AP), immunoprecipitation (IP), sucrose cushion ultracentrifugation (SCU) [24], density gradient centrifugation (DGC), and polymer-based precipitation (PBP) method also known as pellet-down method [25]. The SEC method is based on the differential elution profiles of particles of different sizes running through a porous polymer (gel filtration matrix). Small particles, such as free proteins or protein aggregates, are slowed down when entering the polymer’s pores, while EVs travel quicker along the column and elute first [26,27]. Compared to other methods, SEC could separate different EV subtypes and proteins with minimal effect on the composition and structure of EVs [28]. In the present study, we used the SEC method to separately collect different EV fractions from macrophage-like cells, such as large, medium and small EVs and investigated EV size distribution, morphology under TEM, EV markers (CD9, CD63, and two homologs of HSP90-α and β), molecular transfer to cancer cells, and the alteration of viability in the receiver cancer cells.

The visualization of EVs enables the dissemination of the vesicles in vitro and in vivo to be tracked. Palmitoylation is the covalent attachment of fatty acids to cysteine (S-palmitoylation) and less frequently to serine and threonine (O-palmitoylation) residues of membrane-associated proteins. The cellular and vesicular membrane can be labeled with palmitoylation signal (Palm)-tagged fluorescent proteins [3,24]. We have developed stable cell lines that express Palm fused with green fluorescent proteins (palmGFP) and with tandem dimer Tomato (palmTomato), which visualize the membrane of the cells and their EVs [20]. The uptake of EVs by recipient cells is also visible and quantitative using the palm fluorescent EV system [3]. However, it is uncertain how differently monocyte or macrophage-derived EVs are transmitted to cancer cells. Therefore, in the present study, we investigated the EV-based molecular transfer of palmGFP from macrophages to oral carcinoma cells.

Tumor-derived EVs were able to modulate the tumor microenvironment to foster tumorigenesis, tumor progression, and metastasis. Tumor-derived EVs often contain HSPs [19,29]. We have shown that metastatic oral cancer and metastatic prostate cancer secrete high levels of HSP90-positive EVs and EV-free HSP90, while the targeted knockdown of HSP90α, HSP90β, and their cochaperone CDC37 powerfully diminish EV-driven malignancy events and macrophage M2 polarization in oral cancer [17,19,20,30]. At the same time, HSP90-positive TAMs accumulate in the metastatic oral cancer tissues and infiltrate the tumors [20].

However, macrophage-derived EVs heterogeneity and their effects on receiver cancer cells have not been well understood. In the present study, our aims were as follows: (i) to separate different sized EVs (such as small exosomes, large exosomes, and large EVs) from macrophage-like cells, (ii) to investigate the EV-based molecular transfer of palmGFP (approximately 30kD) to receiver oral carcinoma cells, and (iii) to examine whether the macrophage-EVs altered the cell viability of receiver oral carcinoma cells.

## 2. Materials and Methods

### 2.1. Cells

HSC-3 (human oral squamous cell carcinoma cell line) and THP-1 (human monocytic leukemia cell line) were obtained from JCRB Cell Bank. HSC-3 cells were maintained in D-MEM (Wako, Osaka, Japan). THP-1 cells were grown in RPMI-1640 (Wako). All medium was supplemented with a 10% fetal bovine serum (FBS; Thermo Fisher Scientific, Waltham, MA, USA) and a 1% antibiotic-antimycotic solution (Sigma-Aldrich, St. Louis, MO, USA). Cells were kept in a humidified incubator with 5% CO_2_ at 37 °C. The medium was replaced every 2 days. For macrophage differentiation, THP-1 cells were treated with 20 nM phorbol 12-myristate 13-acetate (PMA) for 24 h or 48 h. Cells were counted using Invitrogen™ Countess™ (Thermo Fisher Scientific, Waltham, MA, USA). Cellular photomicrographs were taken using a Floid Cell Imaging Station (Thermo Fisher Scientific).

### 2.2. EV Fractionation (SEC-CF and PBP)

For the extraction of EVs, the size exclusion chromatography (SEC) combined with the concentration filter (SEC-CF) method and the modified polymer-based precipitation (mPBP) method were used as charted. THP-1 or palmGFP-labelled THP-1 cells (described in Section 2.6) were seeded with 1 × 10^6^ cells in a 100-mm dish and cultured for 24 h. Cells were then treated with 20 nM PMA for 24 h or untreated. The old medium was removed and replaced with 4 mL of serum-free fresh medium, and the cell culture supernatant was collected 48 h after the medium changing. The cell culture supernatant was centrifuged at 2000× *g* for 30 min at 4 °C to remove the detached cells, and then the supernatant was centrifuged at 10,000× *g* for 30 min at 4 °C to remove cell debris [31].

Before the SEC method, the supernatant was concentrated to 500 μL using an ultrafiltration filter (Amicon^®^ Ultra-15 Centrifugal Filter Unit (15 mL/100 kD, Merck Millipore, Burlington, MA, USA) at 5000× *g* and 4 °C. The pass-through (less than 100 kD) was used as a non-EV fraction. The concentrate (500 μL) was applied to the SEC column (qEV original/70 nm, Izon, Christchurch, New Zealand) following the column equilibration procedure according to the manufacturer’s protocol [22]. The sample was eluted with PBS. Twenty fractions of the 500 μL solution pass-through were collected in order. We first divided the fractions into three major parts (fraction 1–6, 7–9, and 10–20), each concentrated 15 times using ultrafiltration devices (Amicon Ultra-0.5 Centrifugal Filter Unit (0.5 mL/100 kD, Merck). Alternatively, we divided the fractions into seven major parts (fractions 1–3, 4–6, 7–9, 10–12, 13–15, 16–18, and 19–21), thereby concentrated them 9 times using ultrafiltration devices (Amicon Ultra-0.5).

For the mPBP method, the supernatant was filtrated with a 0.2-micrometer syringe filter as described [30]. Then the pass-through was concentrated using an ultrafiltration filter (Amicon^®^ Ultra-15 Centrifugal Filter Unit (15 mL/100 kD; Merck) at 5000× *g* and 4 °C. The concentrate was incubated with a Total Exosome Isolation Reagent (Thermo Fisher Scientific) at 4 °C overnight and then centrifuged at 10,000× *g* for 60 min for EV precipitation. The pellet was diluted in 100 μL PBS.

Protein concentrations of the EV fractions were analyzed using a micro-BCA protein assay (Thermo Fisher Scientific).

### 2.3. Particle Size Distribution

We applied 40 μL of each concentrated fraction to a disposable solvent-resistant micro cuvette (ZEN0040, Malvern Panalytical, Malvern, UK). Particle diameters of the fractions in a range between 0 and 10,000 nm were analyzed in a Zetasizer nano ZSP (Malvern Panalytical) using Zetasizer Software ver.7.03.

### 2.4. Transmission Electron Microscopy

A 400-mesh copper grid coated with formvar/carbon films was hydrophilically treated. The concentrated fraction (10 μL) was placed onto a Parafilm, and the grid was floated on the fraction and left for 15 min. The sample was negatively stained with a 2% uranyl acetate solution for 2 min. EVs on the grid were visualized with 10,000 times magnification using an H-7650 transmission electron microscope (TEM) (Hitachi, Tokyo, Japan) at the Central Research Laboratory, Okayama.

### 2.5. Western Blotting

The EV and non-EV fractions were prepared as described above. The first SEC-based gathered fraction sets (Fr. 1–6, 1.51 µg/30 µL; Fr. 7–9, 3.47µg/30 µL; and Fr. 10–20, 58.54 µg/30 µL), PBP-EVs (30 µg/30 µL), or a non-EV fraction (30 µg/30 µL) were mixed with 5 µL of a 6x SDS sample buffer with 9% β-mercaptoethanol and boiled at 95 °C for 5 min. We applied 30 µL of each mixture to 8%, 10% or 12% polyacrylamide gels for sodium dodecyl sulfate polyacrylamide gel electrophoresis (SDS-PAGE).

For second SEC-based gathered fraction sets, 20 µL of each gathered fraction (Fr. 1–3, Fr. 4–6, Fr. 7–9, Fr. 10–12, Fr. 13–15, Fr. 16–18, Fr. 19–21) (see a later figure for protein concentrations), Fr. 1–21 (concentrated CM before SEC) or whole cell lysate (WCL) of macrophages was mixed with 5 µL of a 4x SDS sample buffer without any reducing agent, and boiled at 95 °C for 5 min. We applied 20 µL of each mixture to SDS-PAGE (8%, 10% or 12%).

Proteins in the gel were transferred to PVDF membranes using a wet method. The membranes were blocked in a Tris-buffered saline (TBS) containing 0.05% Tween 20 (TBS-T) and 5% Skim Milk (Fujifilm, Tokyo, Japan) for 1 h with shaking at room temperature (RT). Each membrane was incubated overnight with shaking at 4 °C with the following primary antibodies: rabbit anti-GFP (1:2000, MBL, Tokyo, Japan), rabbit anti-CD9 (1:2000, Abcam, Cambridge, UK), rabbit anti-CD63 (1:1000, System Biosciences, Palo Alto, CA, USA), rabbit anti-HSP90α (1:5000, GeneTex, Irvine, CA), rabbit anti-HSP90β (1:1000, GeneTex), and rabbit anti-actin (1:200, Sigma-Aldrich) antibodies. Afterward, an anti-rabbit IgG secondary antibody conjugated with HRP (1:2500, Cell Signaling Technologies, Danvers, MA, USA) was incubated with the membrane for 1 h on a shaker at RT. Membranes were washed three times before and after the antibody reactions in TBS-T for 10 min at RT on a shaker. Blots were visualized using an ECL Plus Western blotting substrate (Pierce, Rockford, IL, USA).

### 2.6. Fluorescence-Labeling of Cells and EVs

The fluorescent EV reporter constructs were kindly gifted by Dr. Charles P. Lai. Briefly, the lentiviral reporter constructs of CSCW-palmitoylation signal-tandem dimer Tomato (palmT) and CSCW-palmitoylation signal-EGFP (palmG) wer as described [3,20]. PalmG and PalmT sequences were inserted into a SCSGW2 lentivector. For virus production, HEK293T cells were transfected with PalmG or PalmT constructs, psPAX2 packaging plasmid, and pMD2.G envelope plasmid using a polyethyleneimine (PEI) Max transfection reagent (Polysciences, Warrington, PA, USA). THP-1 or HSC-3 cells were infected using the spinfection method with a viral solution. Infected/transduced stable cells were selected using puromycin. Single clones were isolated using limiting dilution method. The stable cells were designated as THP1/palmG and HSC3/palmT cells.

### 2.7. EV Molecular Transfer Assay

As donor cells, THP-1/palmGFP cells were seeded with 1 × 10^6^ cells in a 100-mm dish, cultured for 24 h in RPMI-1640 with 10% FBS, and then treated with 20 nM of PMA for 24 h or untreated. The cells were washed twice with PBS, and then the culture medium was replaced with a serum-free medium and cultured for a further 2 days. The culture supernatant was collected and centrifuged at 2000× *g* for 30 min at 4 °C to remove the detached cells. Then, the supernatant was concentrated ten-fold using an Amicon Ultra-0.5 Centrifugal Filter Unit and used as a CM.

For qualitative analysis, fluorescence images were taken using a confocal laser scanning microscopy (CLSM) system (LSM 780 META, Carl Zeiss, Oberkochen, Germany), as described before [20,32]. Briefly, recipient cells were seeded on a type I collagen-coated coverslip in a 24-well plate at a density of 2.2 × 10^4^ cells per well and cultured for 24 h in D-MEM with 10% FBS.

For quantitative analysis, the fluorescence intensity of the transferred palmGFP from the EV fractions or CM was measured using an ArrayScan High Content Screening (HCS) System ver.6.4.1 (Thermo Fisher Scientific) with channel 485/549/bright field, as described previously [3,32,33]. Briefly, as recipient cells, the HSC-3/palmTomato cells were seeded with 5000 cells per well in a 96-well NanoCulture Plate (NCP) (Medical & Biological Laboratories, Nagoya, Japan) or a 96-well culture plate (Greiner, Kremsmunster, Austria) and cultured for 24 h in 200 μL of mTeSR1 medium or D-MEM. A CM was applied in a ratio of 1:1 with a fresh culture medium. Data represented the total green flurescent signal of all cells in one well.

For the quantitative analysis of the molecular transfer of palmGFP from the EV fractions, HSC-3/palmTomato cells were seeded with 2500 cells per well in a 96-well culture plate (Greiner). After culturing for 24 h in 100 μL of D-MEM, 20 μL of the fractions or PBS (control) was applied to the cells. Data represented the total green flurescent signal of all cells in one well.

### 2.8. Cell Viability

Cell viability was measured as described previously [34]. The ATP content was quantified using a CellTiterGlo (CTG) Luminescent Cell Viability Assay (Promega, Madison, WI). To examine the effects of the CM, whole EV, and co-culture, HSC-3 cells were seeded with 2 × 10^4^ cells per well in a 24-well plate. After a 24 h of culture period, the following was carried out for the CM and co-culture, respectively: (1) an equal volume of a CM was added to the culture medium, or (2) a co-culture insert with a 0.45-micrometer pore (Greiner, Kremsmunster, Austria) was placed, and THP-1 cells (2 × 10^4^ cells/well) were seeded in the co-culture insert. After culturing for another 48 h, cells were detached using Trypsin/EDTA and suspended with a 150 μL of CTG solution and then incubated for 10 min at 37 °C. Luminescence was measured using the plate reader Gemini XP SOK SoftMax Pro ver.5.3 (Molecular Devices).

To examine the effects of the EV fractions separated using the SEC method, HSC-3 cells (5000 cells/well/200 μL) were seeded into a 96-well plate. After 24 h of culturing, 10 μL of the EV fractions (Fr. 1–6, 7–9, or 10–20) were added to each well (quadruplicate). After culturing for another 48 h, 150 μL of medium was removed from each well, and 50 μL of CTG solution was added. Luminescence was measured using the Gemini XP SOK.

In the case of the gathered EV fractions (Fr. 1–3, 4–6, 7–9, 10–12, 13–15, 16–18, and 19–21), HSC-3 cells (2,500 cells/well/100 μL) were seeded into a 96-wells plate. After the incubation for 24 h, 15 μL of the EV fractions were added to each well (triplicate). After culturing for another 48 h, 100 μL of CTG solution was added. Luminescence was measured using the Gemini XP SOK.

### 2.9. Flow Cytometry

The following phycoerythrin (PE)-conjugated mouse monoclonal anti-human antibodies were used for cell surface staining: anti-CD14, anti-CD80, anti-CD206, and anti-CD68 antibodies (all from BioLegend, San Diego, CA, USA). Isotype-matched negative controls (CD14, mouse IgG2aκ; CD68, mouse IgG2bκ; CD80 and CD206, mouse IgG1κ) were used throughout the investigations. In negative control groups, the cells were not treated with an antibody. THP-1 cells and PMA-induced macrophages were cultured in a serum-free medium for 48 h. PMA-induced macrophages were unattached with a Accutase (Innovative Cell Technologies, San Diego, CA, USA) and centrifuged at 200× *g* for 5 min at 4 °C. The pellet was washed with PBS containing 0.5% bovine serum albumin (BSA, Wako). Fc receptors were blocked by incubating the cells with a 25 μg/mL of Fc receptor blocking solution (Human TruStain FcX™, BioLegend) for 10 min at RT before antibody staining. To detect cell surface markers, 5 μL of monoclonal mouse anti-human antibodies or the relevant isotypes were incubated with the samples for 30 min at 4 °C. For intracellular staining (CD68), cells were fixed with a 4% paraformaldehyde (PFA) phosphate buffer solution (Wako) and permeabilized with Nonidet P-40 (NP-40; Sigma-Aldrich). The stained cells were analyzed using a MACSQunat^®^X flow cytometer (Miltenyi Biotec, Bergisch Gladbach, Germany), and more than 4000 events were measured per sample. The flowcytometric analyses were carried out using MACSQunat ver.2.6 software (Miltenyi Biotec). All experiments were performed in triplicate.

### 2.10. Statistical Analysis

Statistical significance was calculated using GraphPad Prism ver.8.0.1 and Microsoft Excel. The difference between the two sets of data was examined using an unpaired student’s *t*-test or a one-way ANOVA. The difference between the three sets of data was examined using a two-way ANOVA using GraphPad Prism 8.0.1. *p* < 0.05 was considered to indicate statistical significance. Data were expressed as means ± SD unless otherwise specified.

## 3. Results

### 3.1. Differentiation of Monocytic Cells into Macrophage-Like Cells

**Figure 1:**
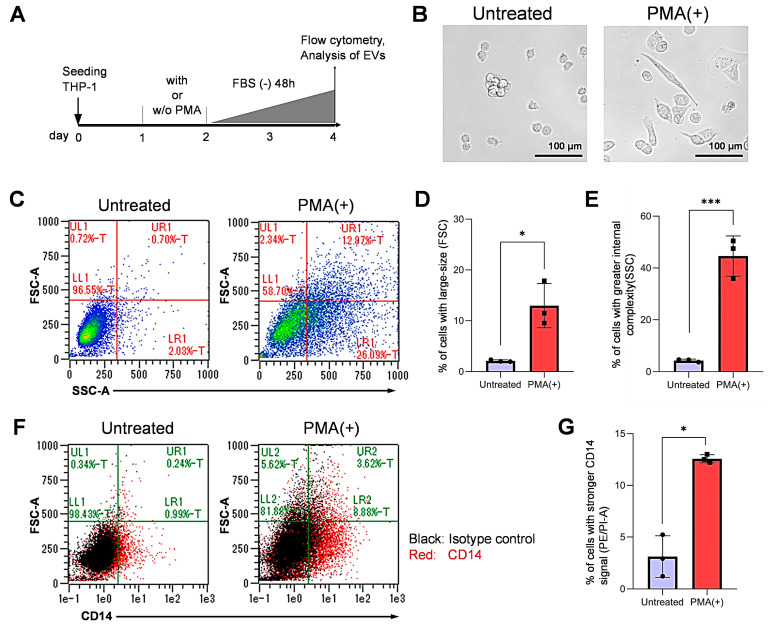
Differentiation of macrophage-like cells from monocytic cells. (**A**) A scheme of experimental protocols. THP-1 cells were stimulated with PMA for 24 h or unstimulated. (**B**) Representative images of PMA-treated and non-treated THP-1 cells. Scale bars, 100 µm. (**C**) flow cytometry showing size (FSC) and complexity (SSC) of the PMA-treated and the non-treated THP-1 cells. Data are representative of three independent experiments. See Figure S1A for replicate data. (**D**) Enlargement of cell size upon PMA treatment. N = 3 (biological triplicate), * *p <* 0.05 (**E**) Increased internal complexity of cells upon PMA treatment. N = 3 (biological triplicate), *** *p <* 0.001 (**F**) Flow cytometry detecting cell surface CD14. Data are representative of three independent experiments. See Figure S1B for replicate data. (**G**) percentages of the CD14-positive cells obatained from flow cytometry. N = 3 (biological triplicate), * *p <* 0.05. See Figure S1C for analysis of M1 and M2 markers.

We first examined whether PMA stimulation of THP-1 monocytic cells could foster macrophage differentiation into M0, M1, or M2 -types. For this purpose, we examined the size and shape of the non-treated THP-1 and the PMA-stimulated THP-1. Cellular attachment and enlargement appeared to be increased after PMA stimulation (Figure 1A,B). Indeed, the percentage of large-sized cells (vertical axis, FSC) was significantly higher in the PMA-stimulated THP-1 (upper left (UL) zone + upper right (UR) zone = 15.31%) compared to the non-treated cells (1.42%) Figure 1C,D and Figure S1A). The internal complexity (i.e., granularity) (horizontal axis, SSC) were greater in the PMA-stimulated THP-1 (UR+LR, 39.06%) than the non-treated THP-1 (2.73%) (Figure 1C,E).

We next examined CD14, one of established macropahge markers by flow cytometry. The percentage of CD14-positive cells was significantly increased from approx. 1% to 12% upon PMA treatment, indicating that cells were differentiated to macrophage-like cells (Figure 1F,G and Figure S1B). To characterize the macrophage subtypes such as M0, M1, or M2 types, we next examined the expression of CD68, CD80, and CD 206 in the untreated vs. the PMA-stimulated THP-1 cells. Meanwhile, intracellular and cell-surface CD68 (markers of pan-macrophages), CD80 (a marker of M1 macrophages), and CD206 (a marker of M2 macrophages) did not differ from their isotype controls (Figure S1C).

These data indicate that the PMA-stimulated THP-1 monocytic cells were differentiated into macrophages but neither of M1 nor M2 types.

### 3.2. High Transmission Efficiency of Macrophage-Derived EVs to Oral Carcinoma Cells

**Figure 2:**
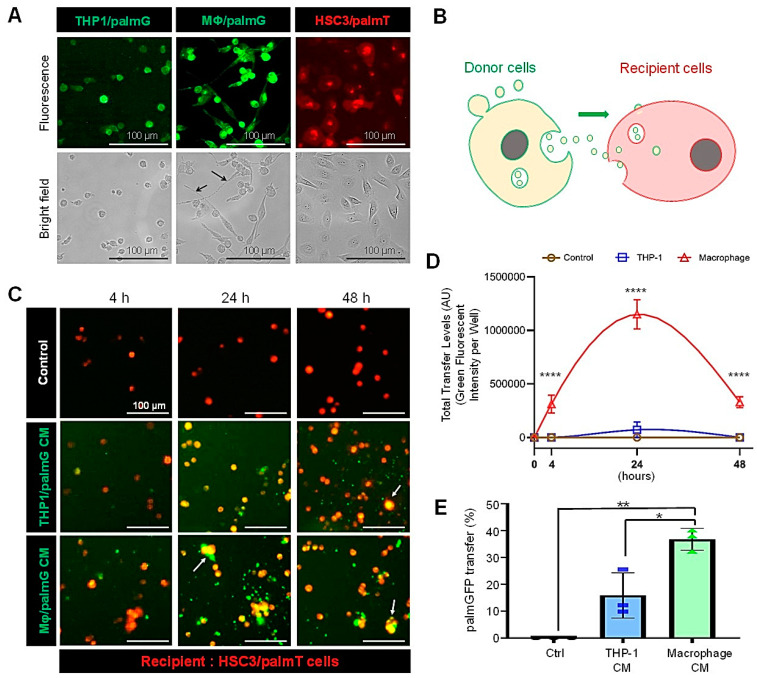
Molecular transfer of palmGFP from macrophage-like cells to oral carcinoma cells. Palmitoylation signal-fused with enhanced GFP (palmG) (shown as green) and with tdTomato (palmT) (shown as red) were stably expressed in the THP-1 or HSC-3 cells respectively, and designated as THP1/palmG and HSC3/palmT cells. The THP1/palmG cells were stimulated with PMA for a macrophage-like differentiation. (**A**) Representative images of the THP1/palmG, Macrophage/palmG, and HSC3/palmT cells. The arrows indicate the dendritic projections formed upon differentiation. (**B**) The concept of EV transfer analysis. (**C**,**D**) EV transfer assay in a 3D culture system. The recipient HSC3/palmT cells (red) were seeded with 5000 cells per 200 µL of mTeSR1 per well in a 96-well NanoCulture Plate (NCP) and cultured for 24 h. Then, 100 µL of the medium was removed carefully, and 100 µL of a conditioned medium (CM) of macrophage/palmGFP cells or THP1/palmGFP cells was added. In the control group, serum-free RPMI-1640 media were added. (**C**) Representative images of EV transfer. Images were taken after 4, 24, or 48 h of the CM addition. See Figure S2 for full images. (**D**) Total transfer levels of palmGFP per well. AU, arbitrary unit. N = 3 (biological triplicate), **** *p <* 0.0001 (THP-1 vs. Macrophages at 4, 24, and 48 h) (**E**) Transfer efficiencies of palmGFP from a CM in the 2D culture system 24 h after the CM addition. N = 3 (biological triplicate), * *p <* 0.05, ** *p <* 0.01. See Figure S4 for representative images of palmGFP transfer from the CM.

To monitor macrophage-derived EVs’ uptake by oral cancer cells, we labeled the membrane of the THP-1 cells and their EVs with palmGFP and of HSC-3 cells with PalmTomato. Then, we confirmed that the palm-fluorescent proteins were successfully expressed in these cells (Figure 2A). Besides, THP-1/palmG cells were differentiated by stimulation with PMA to macrophage-like cells expressing palmGFP, which were more attached to the dishes and larger in size (Figure 2A, arrows).

Next, we examined the molecular transfer of palmGFP from the macrophage-like cells or monocytic cells to the PalmTomato/HSC3 oral carcinoma cells in a 3D culture system. PalmGFP appeared to be transferred from the conditioned media of both macrophages and monocytic cells to cell aggregates of PalmT/HSC3 (Figure 2C and Figure S2). (Since the labelling efficiency of PalmT/HSC3 was not 100%, some palmGFP still transferred into these unlabelled cell aggregates.) The macrophage-secreted palmGFP was more efficiently transferred to receiver cells than the monocyte-derived palmGFP after 4, 24, and 48 h of CM treatment (Figure 2D). The molecular trasnfer level of the palmGFP from macrophages was 15.8-fold higher than from THP-1 cells at 24 h.

Next, we confirmed the transfer of macrophage-derived palmGFP to macrophages themselves (Figure S3) and oral cancer cells in a 2D culture under confocal laser scanning microscopy (Figure S4). Then, we examined the molecular transfer efficiency of palmGFP into oral carcinoma cells in a 2D culture environment. The molecular transfer efficiency of palmGFP in a macrophage-CM to oral cancer cells was significantly higher than in a monocyte-CM (Figure 2E). The monocyte-palmGFP was transferred to 15.8% of the recipient HSC-3 cells, while the macrophage-palmGFP was transferred to 36.7% of the recipient cells (Figure 2E).

These data indicated that the macrophage-derived palmGFP was highly transferrable to receiver oral carcinoma cells compared to monocyte-derived palmGFP, suggesting that EVs enclosing palmGFP could alter molecular transfer efficiencies.

### 3.3. Macrophage-Secreted Factors Reduced Cell Viability in Receiver Oral Carcinoma Cells

**Figure 3:**
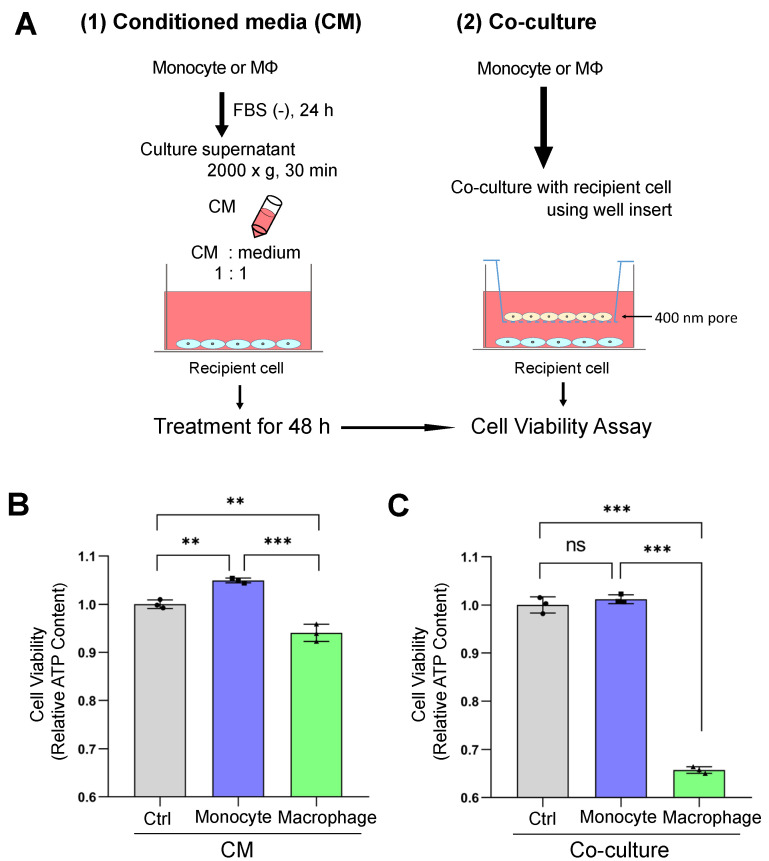
Macrophage-secreted factors lowered the cell viability of oral carcinoma cells. (**A**) Schemas of experimental designs. (1) HSC3/palmTomato cells were treated with a CM collected from THP1/palmGFP or macrophage/palmGFP cells for 48 h. (2) THP1/palmGFP or macrophage/palmG cells were co-cultured with HSC3/palmTomato cells using a well-insert (400 nm pore) for 48 h. (**B**) Cell viability of HSC3/palmTomato cells treated with a CM. (**C**) Cell viability of HSC3/palmTomato cells co-cultured with the THP1/palmGFP or macrophage/palmGFP cells. N = 3 (biological triplicate), ** *p <* 0.01 and *** *p <* 0.001. See Figures S3 and S4 for confocal images of palmGFP transfer.

We next established intercellular communication experiments using a conditioned medium (CM) and a transwell-based co-culture system (Figure 3A). To ask whether macrophage-secreted factors alter the recipient cell’s viability, we examined the ATP content of the HSC-3 cells after receiving macrophage-secreted factors in the CM or in the co-culture system. The macrophage-CM significantly reduced the ATP content of HSC-3 cells (Figure 3B), whereas the monocyte-CM increased the ATP content. The ATP content of HSC-3 cells after co-culturing with macrophages was reduced to 65% (Figure 3C), whereas co-culturing with monocytes did not alter the ATP content.

These data suggest that macrophage-secreted factors decreased the viability of oral carcinoma cells.

### 3.4. Separation of Large, Medium, and Small EVs Using the SEC-CF Method

**Figure 4:**
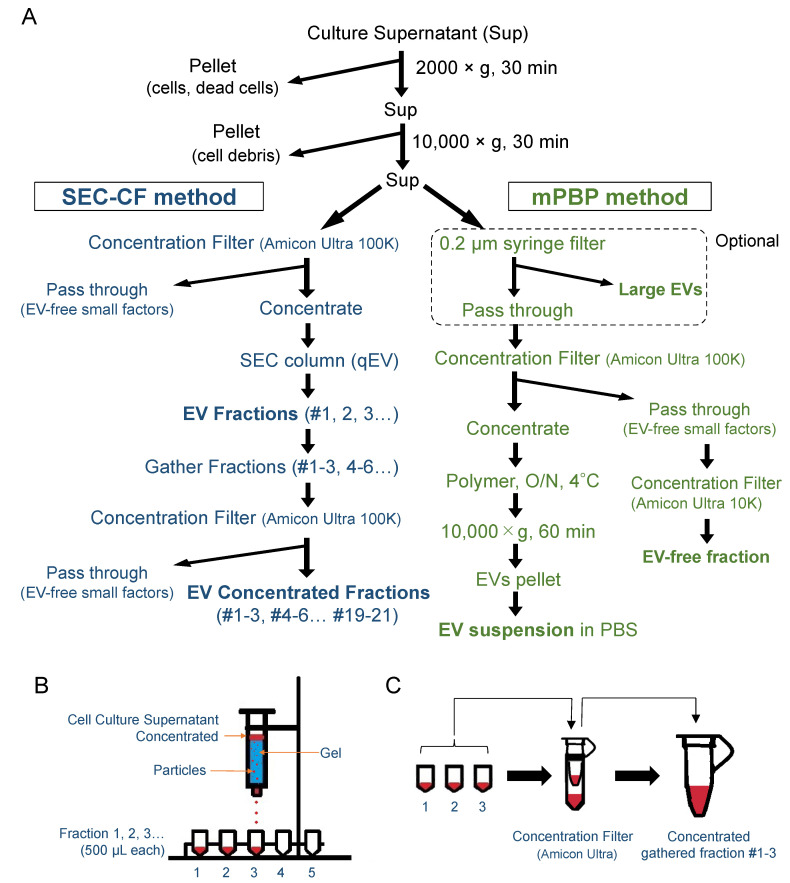
Preparation of EV fractions using size exclusion chromatography with the concentration filter (SEC-CF) method and the modified polymer-based precipitation (mPBP) method. (**A**) A flow chart of the SEC-CF method and the mPBP method. (**B**) A scheme of the SEC method. (**C**) A scheme of the filtration concentration step useful to concentrate gathered fraction sets.

The heterogeneity of EVs with different marker proteins could lead to a diversity in EV functions [35]. To ask how different EV types were isolated using different methods, we next used the SEC-CF method and the mPBP method (Figure 4A). Using the SEC method, we first fractionated the culture supernatant into 20 fractions (Fractions 1, 2, 3…20) (Figure 4B). To simplify the EV analysis, we gathered these fractions into the following three groups using concentration filters: fraction 1–6 (Fr. 1–6), fraction 7–9 (Fr. 7–9), and fraction 10–20 (Fr. 10–20) (Figure S5 and Figure 4C).

**Figure 5:**
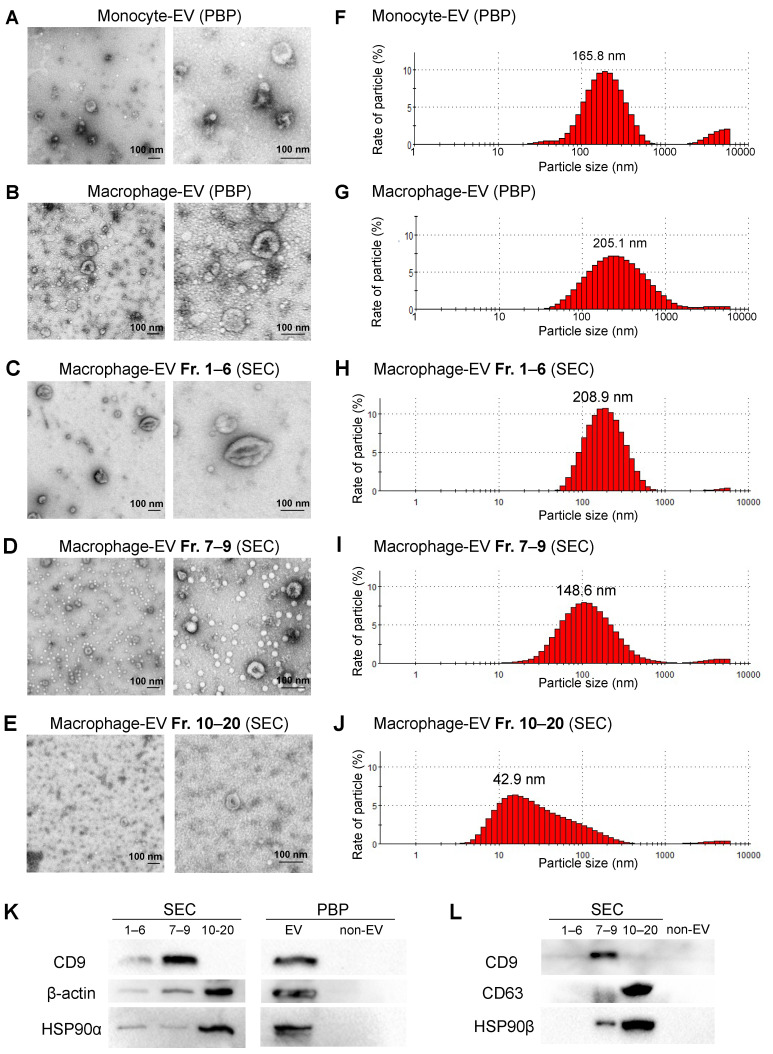
SEC-CF-based characterization of the small, medium, and large EVs released by macrophage-like cells. Conditioned medium was collected from (**A**) untreated THP-1 monocytic cells or (**B**–**E**) PMA-treated macrophage-like cells 48 h after changing to a serum-free medium. (**C**–**E**) Twenty fractions were first collected using the SEC method and then concentrated into three fractions (Fr.1–6, Fr.7–9, and Fr.10–20) using concentration filters. See details in Figure S5. (**A**–**E**) Representative TEM images of the EVs derived from (**A**) monocytic cells and (**B**–**E**) macrophages. Scale bars, 100 nm. See Figures S6 and S7 for full images of TEM. (**F**–**J**) Particle diameter distribution of the EV fractions. (**K**) Western blotting of CD9, HSP90α, and β-actin. (**L**) Western blotting of CD9, CD63, and HSP90β. See Figures S8 and S9 for full images. See Figure S10 for particle diameter distribution.

The cup-like shapes were observed in the EVs released from both the macrophage and monocyte using TEM, indicating that both cell types secreted exosome-like EVs (Figure 5A–E and Figures S6 and S7). The size of the macrophage-EVs appeared to be larger and more varied than the monocyte-EVs (Figure 5A,B,F,G). In Fr. 1–6, a few “large EVs”, with a size of approximately 200–400 nm, were observed using TEM (Figure 5C and Figure S7). In Fr. 7–9, medium EVs (50–150 nm) were observed under a TEM (Figure 5D and Figure S7), supposed to be large exosomes (EXO-L) based on their size. Notably, Fr. 10–20 contained fewer small EVs (approximately 50 nm) (Figure 5E and Figure S7), suggesting that Fr. 10–20 could contain small exosomes (EXO-S).

Next, particle diameter distribution analysis revealed that the size of monocyte-EVs peaked at 165.8 nm in the range between 50–500 nm, while that of macrophage-EVs peaked at 205.1 nm in the wider range between 50–1000 nm (Figure 5F,G), consistently with the TEM data, suggesting that macrophage-EVs tended to be larger and more various than monocyte-EVs.

In Fr. 1–6 of the SEC method, the particle size was between 100–500 nm and peaked at 208.9 nm (Figure 5H), suggesting that Fr. 1–6 contained large EVs (larger than exosomes). In Fr. 7–9, the particle size ranged between 50–300 nm with a peaked size of approximately 150 nm (Figure 5I), suggesting large exosomes (EXO-L) from the size. In Fr. 10–20, the size of particles was smaller than 100 nm and peaked at approximately 40 nm (Figure 5J), consistent with the TEM data. Thus, Fr. 10–20 could contain small exosomes (EXO-S).

To characterize the small and large exosomes or larger EVs using protein markers, we next performed Western blotting of tetraspanins (CD9 and CD63 are established EV markers), HSP90α, HSP90β (often found in EVs), and β-actin. CD9 was markedly detected in the EXO-L fraction (Fr. 7–9) (Figure 5K,L, Figures S8 and S9). On the other hand, CD63, another tetraspanin family member often found in EVs, was markedly detected in the Fr. 10–20 (EXO-S) while the degradation of CD63 was seen in Fr. 7–9 (Figure S9), suggesting that CD63 might be selectively cleaved out from the EXO-S by metalloproteinases [3,32]. HSP90α, HSP90β, and β-actin were markedly found in Fr. 10–20 (Figure 5K,L), suggesting that these HSP90 homologs and β-actin mainly existed in the EXO-S fraction. Additionally, HSP90β was detectable in the EXO-L fraction (Fr. 7–9) while it was not contained in the large EV fraction.

These data indicate that different EV types can be separately isolated using the SEC method as EXO-L, EXO-S, and large EVs. These data also suggest that macrophages released CD9-positive large exosomes (EXO-L) and CD63/HSP90-positive small exosomes.

### 3.5. Macrophage-Secreted Factors (Fraction 10–20 Containing Small Exosomes) Decreased Viability of Oral Carcinoma Cells

**Figure 6:**
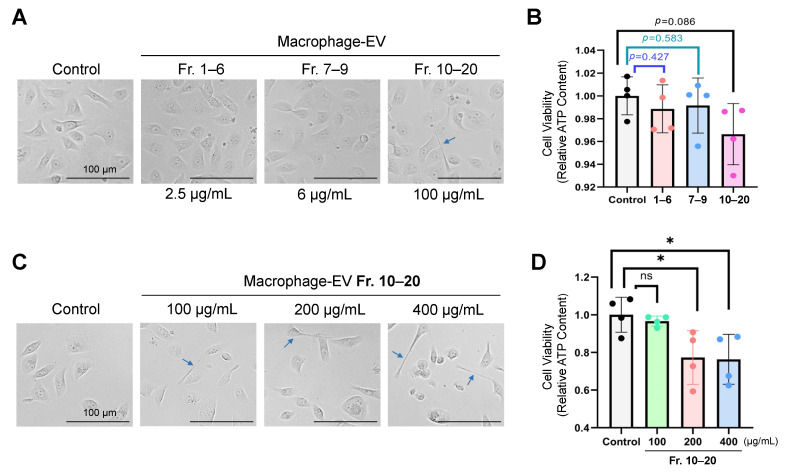
Macrophage-secreted small EV fraction (Fr. 10–20, approximately 10–70 nm) reduced the cell viability of oral carcinoma cells. EV fractions were prepared from THP-1-derived macrophages using the SEC-CF method and administrated to HSC-3 cells for 48 h. The ATP content was measured to determine cell viability. (**A**,**B**) HSC-3 cells were treated with macrophage-EVs Fr. 1–6, 7–9, or 10–20. PBS was added to the control group. (**A**) Representative images of the HSC-3 cells treated with the fractions (10 µL each) for 48 h. Final concentrations of the EV fractions were shown. Scale bars, 100 µm. Arrows indicate a projection from a cell. (**B**) Relative ATP content of the recipient HSC-3 cells treated with the fractions. N = 4 (biological quadruplicates). (**C**,**D**) HSC-3 cells were treated with macrophage-EVs Fr. 10–20 at the final concentrations of 100, 200, or 400 µg/mL for 48 h. PBS (40 µL) was added to the control group. (**C**) Representative images of the HSC-3 cells treated with Fr. 10–20. Scale bars, 100 µm. Arrows indicate projections from spindle-like cells. (**D**) Relative ATP content of the HSC-3 cells treated with Fr. 10–20. Relative values of the control group were plotted. N = 4 (biological replicates). * *p <* 0.05. n.s., not significant.

To examine whether macrophage-EVs alter the viability of recipient oral carcinoma cells, we stimulated the HSC-3 cells with the EV fractions (Fr. 1–6, 7–9, and 10–20) and then measured the ATP content. The morphology of the HSC-3 cells was changed to spindle-like shapes after applying Fr. 10–20 (EXO-S) but Fr.1–6 nor Fr. 7–9 (Figure 6A). The EV Fr. 10–20 tended to lower the viability of the HSC-3 cells (*p* = 0.0863), which was more effective than the other two fractions (Figure 6B). (Note that the protein concentrations of the EV fractions were largely different, as shown in Figure S5).

We next examined the EV effects at three different concentrations (100 µg/mL, 200 µg/mL, and 400 µg/mL) of Fr. 10–20 on the oral carcinoma cells. The spindle shapes were found in the HSC-3 cells stimulated with Fr. 10–20 at these concentrations, whereas they were not found in the unstimulated cells (Figure 6C). Simultaneously, the cell viability of HSC-3 cells was reduced to approximately 77% after applying the higher concentrations (200 µg/mL and 400 µg/mL) of the EV Fr. 10–20 (Figure 6D).

These data indicate that macrophages-derived EXO-S decrease the viability of oral carcinoma cells.

### 3.6. Size-Based Fractionation of Macrophage/palmGFP-Secreted EVs

**Figure 7:**
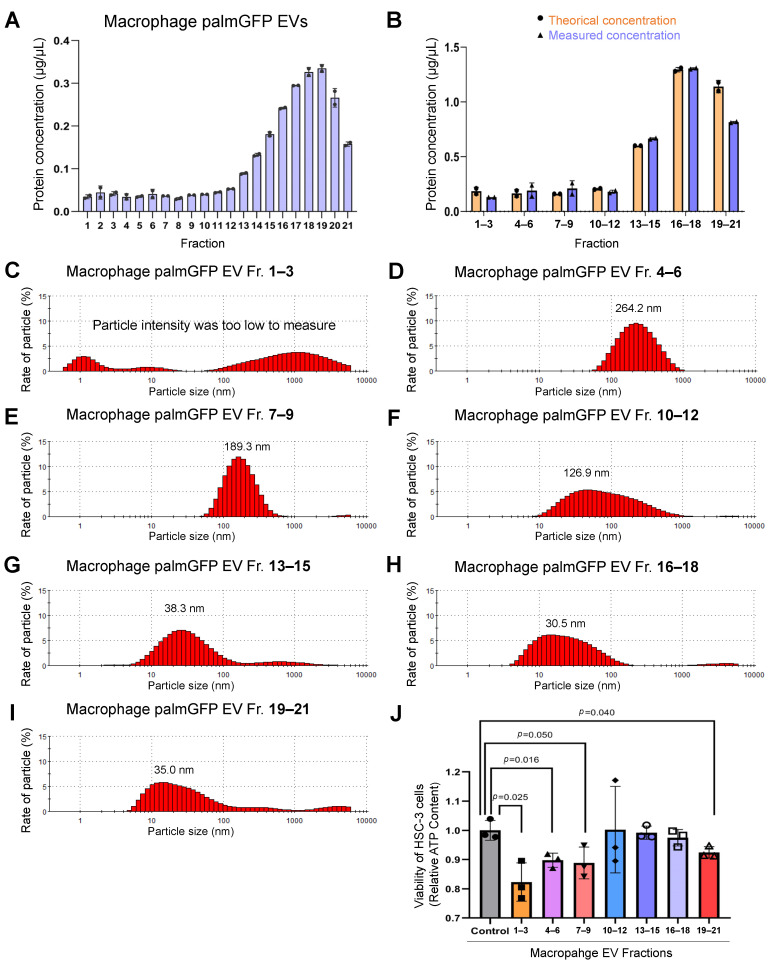
Size exclusion chromatography and concentration filtering (SEC-CF) to analyze the macrophage-EVs that reduced the cell viability of oral carcinoma cells. (**A**–**J**) A CM of macrophage/palmG cells was fractionated. (**A**) Protein concentrations of each fraction. N = 2 (technical replicate) (**B**) Protein concentrations of the gathered fractions (Fr. 1–3, Figure 4. Fr. 7–9, Fr. 10–12, Fr. 13–15, Fr. 16–18, and Fr. 19–21). Theoretical concentrations were calculated from the average of three fractions and the concentration rate based on the data shown in Figure 7A. N = 2 (technical replicate). (**C**–**I**) Particle diameter distribution of macrophage/palmG EV fractions. (**J**) Macrophage-EV fractions reduced the ATP content in the receiver HSC-3 cells. HSC-3 cells were seeded with 2500 cells per 100 µL per well in a 96-well plate. After culturing for 24 h, 15 µL of each fraction was added to receiver cells. The ATP content was measured 48 h after applying the EV fractions. N = 3 (biological triplicate). *p* values under 0.05 are shown on the graph.

To further examine the characteristics of different EV subtypes, we prepared macrophage/palmGFP EV fractions using the SEC method. We investigated the protein concentrations of each EV fraction and found that fraction 1–12 contained very few proteins (lower than 0.05 µg/µL) while fraction 14–21 showed higher protein concentrations (higher than 0.1 to 0.3 µg/µL) (Figure 7A). To simplify the EV analysis, we gathered these fractions into the following seven fractions: fraction 1–3 (Fr. 1–3), fraction 4–6 (Fr. 4–6), fraction 7–9 (Fr. 7–9), fraction 10–12 (Fr. 10–12), fraction 13–15 (Fr. 13–15), fraction 16–18 (Fr. 16–18), and fraction 19–21 (Fr. 19–21) (Figure 4C). The theoretical protein concentration of these fractions was calculated and compared with the actual measured concentration, and no significant difference was found (Figure 7B).

To examine the size variety of the gathered fractions, we measured the particle diameter distribution. The particle intensity in Fr. 1–3 was too low to be detected accurately (Figure 7C), while some large particles (approximately 500–3000 nm) still could be detected, suggesting that Fr.1–3 may contain very large EVs such as apoptotic bodies. EV size of Fr. 4–6 was peaked at 264.2 nm in the range between 100–500 nm (Figure 7D), suggesting Fr. 4–6 may contain medium EVs. EV Fr. 7–9 was peaked at 189.3 nm in the range between 80–300 nm (Figure 7E), which was similar to CD9-EVs in Figure 5I, also suggesting Fr. 7–9 might contain large EXO-L. In the Fr. 10–12, the peak appeared at 126.9 nm in the wider range between 20–200 nm (Figure 7F), while Fr.13–15, Fr. 16–18, and Fr. 19–21 showed similar peaks at around 30 nm and range from 10–70 nm (Figure 7G–I), which contained CD63 as shown above.

These data indicated that EV subtypes in the gathered fractions were different from each other: Fr. 1–3 contained large EV 500–3000 nm, potentially apoptosomes; Fr. 4–6 contained medium EV 100–500 nm, potentially MVs; Fr. 7–9 contained EXO-L 80–300 nm; Fr. 10–12 contained uncertain particles; Fr. 13–21 contained EXO-S 10–70 nm.

### 3.7. Macrophage-Derived (Large and Small) EVs Reduced Viability of Oral Carcinoma Cells

Since Fr. 1–6, Fr. 7–9, and Fr. 10–20 prepared using the SEC-CF method showed a tendency to reduce the viability of HSC-3 (shown in Figure 6), we further examined whether Fr. 1–3, Fr. 4–6, Fr. 7–9, Fr. 10–12, Fr. 13–15, Fr. 16–18, or Fr. 19–21 of macrophage-EVs could reduce the viability of HSC-3 cells. Fluorescent-labeled EVs could generate a light signal, influencing the reading of fluorescent or luminescent values for cell viability assay. Therefore, we prepared the unlabeled macrophage-derived EV fractions using SEC and concentrated them into seven fractions (Fr. 1–3, Fr. 4–6, Fr. 7–9, Fr. 10–12, Fr. 13–15, Fr. 16–18, and Fr. 19–21). After applying 20 µL of these fractions, the Fr. 1–3 (*p* = 0.025), Fr. 4–6 (*p* = 0.016), Fr. 7–9 (*p* = 0.050), and Fr. 19–21 (*p* = 0.040) significantly decreased the ATP content of the receiver cells as compared to the control (Figure 7J).

These data show that macrophage-derived large and small EVs reduced the viability of oral carcinoma cells.

### 3.8. Molecular Transfer of palmGFP from Macrophage-EVs to Oral Carcinoma Cells

**Figure 8:**
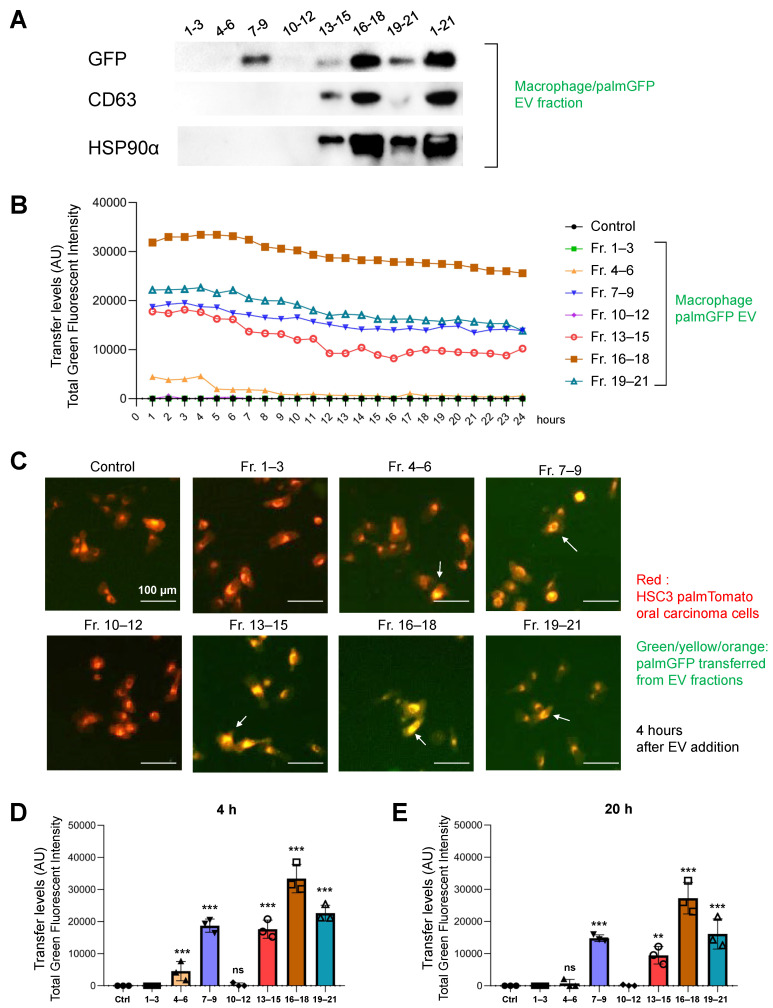
Characterization and molecular transfer of macrophage/palmGFP-EVs. (**A**) Western blotting of GFP, CD63, HSP90α in the macrophage/palmGFP fractions prepared using the SEC-CF method. See applied protein amounts and full images in Figure S11. (**B**) Transfer levels of palmGFP in time course from 1–24 h after applying different fractions. N = 3 (biological replicate). PalmT/HSC3 recipient cells were seeded with 2500 cells per 100 µL per well in a 96-well plate. After culturing for 24 h, 20 µL of the macrophage/palmGFP EV fractions or PBS was added to the receiver cells. The green fluorescent intensity was measured using the ArrayScan system from 1–24 h after applying the fractions. (**C**) Representative merged images of the palmGFP transfer from different EV fractions to HSC3/palmTomato cells. Cells were treated with EVs for 4 h. Arrows indicate the cells that received macrophage-derived palmGFP from EV fractions. See full images in Figure S12. (**D**,**E**) Quantitative analysis of the palmGFP transfer from different EV fractions (**D**) 4 h or (**E**) 20 h after the EV addition. N = 3 (biological replicate). ** *p <* 0.01, *** *p* < 0.001 (compared with a control group). AU, arbitrary unit.

To molecualrly characterize the gathered fractions from the macrophage/palmGFP cells, we carried out western blotting of GFP, CD63, and HSP90α. GFP was detected in the Fr. 7–9 (EXO-L), 13–15, Fr. 16–18, and Fr. 19–21 (EXO-S), suggesting that the EXO-L and EXO-S cotained palmGFP. Indeed, CD63 was detected in Fr. 13–15, Fr. 16–18, and Fr. 19–21 (Figure 8A and Figure S11), suggesting that these fractions contained EXO-S. HSP90α was also detected in the same fractions (Fr. 13–21).

To examine the different molecular transfer activities of EV fractions, we examined the palmGFP transfer from the seven gathered fractions of macrophage/palmG-EVs to oral cancer cells in a 2D culture system. Fr. 7–9 (containing EXO-L), Fr. 13–15, Fr. 16–18, and Fr. 19–21 (containing EXO-S) showed relatively high palmGFP transfer levels to oral cancer cells from 1 to 24 h (Figure 8B), while the palmGFP transfer from Fr. 4–6 (potentially containing MVs) was more rapid in the first 4 h, and then reduced after 5 h. GFP was not detected by adding Fr. 1–3, Fr. 10–12, or the PBS control, which was consistent with the results of western blotting.

A high green fluorescent intensity tended to be captured in the receiver cells upon the addition of Fr. 7–9, Fr. 13–15, Fr. 16–18, and Fr. 19–21 at 4 h using the ArrayScan system (Figure 8C and Figure S12), also indicating that the molecular transfer to oral cancer cells happened at higher levels in these fractions as compared to the other fractions or the control. To ask statistical significance, we next examined the quantitative measure of molecular transfer levels of these EV fractions. We found the Fr. 7–9, Fr. 13–15, Fr. 16–18, and Fr. 19–21 showed a significant molecular transfer at both 4 h and 20 h (Figure 8D,E), while Fr. 4–6 showed a significant molecular transfer only at 4 h but not at 20 h. Fr. 1–3 and 10–12 did not show any significant molecular transfer.

These data indicate that EXO-L (Fr. 7–9, CD9-positive) and EXO-S (Fr. 13–21, CD63/HSP90-positive) involve an efficient molecular transfer from macrophage-EVs to receiver carcinoma cells. In addition, together with the viability data in Figure 6 and Figure 7, the EXO-L and EXO-S might have an ability to reduce the ATP content in receiver carcinoma cells (Table 1).

## 4. Discussion

The heterogeneity of EVs and extracellular particles is currently a crucial issue in biology and medicine. Many methodologies have been developed to separate various EVs according to the surface marker specificity, EV size, and density. The SEC method enables the separation of vesicle and protein fractions according to their size. Indeed, we collected twenty fractions from macrophage CM and then characterized several sets of the fractions. The large EV (500–3000 nm) fraction was rarely collected and reduced the viability of receiver oral carcinoma cells. The medium EV (100–500 nm) fraction, presumably containing shed EVs, rapidly transferred palmGFP to the receiver cancer cells and reduced viability. The CD9-positive EV (80–300 nm) fraction, presumably containing large exosomes, rapidly transferred palmGFP to the receiver cells and reduced the cell viability. Fraction 10–

**Figure 9:**
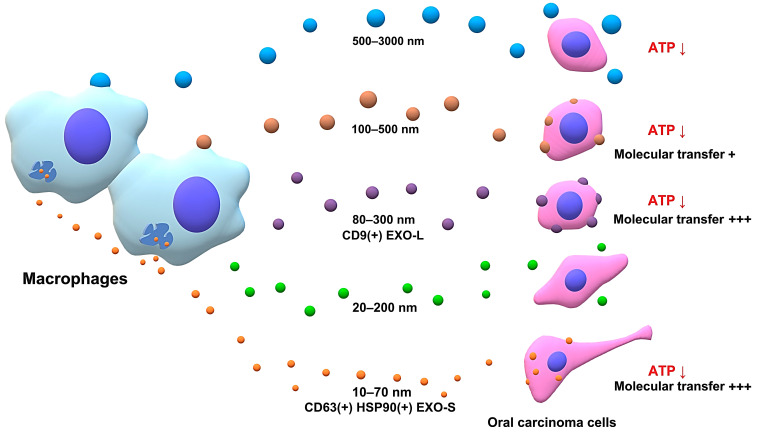
Graphical abstract. Extracellular vesicles are heterogenous in their size, cargos, and functions. The SEC and concentration filter (SEC-CF) method fractionated five EV-types from the culture supernatant of macrophage-like cells. EVs (500–3000 nm) are potentially apoptosomes (shown in blue). EVs (100–500 nm) are potentially microvesicles (shown in brown). EVs (80–300 nm) are CD9-positive large exosomes (EXO-L) (shown in purple). EVs (20–200 nm) are unidentified vesicles/particles (shown in green). EVs (10–70 nm) contain CD63/HSP90-positive small exosomes (EXO-S) (shown in orange). Small and large exosomes showed efficient molecular transfer activities and a reduced ATP content, an indicator of cell viability in receiver carcinoma cells compared to other EV types. The experimental evidence is summarized in Table 1.

12 (EV 20–200 nm) is currently featureless. Small EV/particle fractions (10–70 nm) were protein-rich with CD63, HSP90α, and HSP90β and highly transferred palmGFP to receiver cancer cells and reduced the viability. In the present study, we showed that molecularly transferable EVs (m-EV 80–300 nm and s-EV 10–70 nm) contained tetraspanins (CD9 and CD63) and HSP (HSP90α and HSP90β). Consistently, HSP90, CD9, and CD63 are essential for EV-based molecular transfer [20]. Thus, it is consistent that the high transfer activity of EVs to receiver cells depends on tetraspanins (CD9, CD63) and HSP90 in the EVs. We also detected that macrophage-EV fractions reduced the ATP content in the receiver carcinoma cells, although we have not identified the factors responsible for reducing cell viability, such as microRNA, cytokines, or chemokines. Nevertheless, we fractionated different EV types such as small, medium, and large EVs, with different molecular transfer activities (Table 1, Figure 9).

We separated CD9-positive large exosomes (Fr. 7–9) and CD63-positive small exosomes (Fr. 13–18). The protein markers of EVs have been investigated by many researchers and organized in MISEV2018 [16]. The key members of the tetraspanin family, CD9 and CD63, were usually used as exosomes markers, whereas they were also detected in other types of EVs [36]. EVs consisted of heterogeneous subtypes, among which CD9 was mainly contained in EXO-L (90–120 nm), while CD63 was mainly in EXO-S (60–80 nm) [15], which is consistent with our data. The distinction of EV markers can be explained by their origin because CD9-positive EVs are formed at the plasma membrane and early endocytic locations, while CD63-positive EVs were specifically abundant in proteins associated with endosomes, multivesicular bodies (MVB), vacuoles, and phagocytic vesicles [15,36]. In the present study, EXO-L with a strong CD9 signal was mainly presented in faction 7–9, while EXO-S appeared to be presented only in fraction 10–20. In addition, our data indicated that CD9-EVs and CD63-EVs were highly transferable to receiver cells. Therefore, the SEC-based preparation of EVs can be useful for efficient molecular transfer and delivery in vitro and in vivo.

Moreover, HSP90 homologs were co-detected with CD63 in the small EV fraction (Fr. 10–20). Notably, extracellular HSP90 exists in EVs, on the membrane surface of EVs, and in vesicle-free forms in a context-dependent manner [17,19,20]. HSP90 plays key roles in phagocytosis and antigen cross-presentation of antigen-presenting cells (APC) such as dendritic cells and macrophages [29,37,38]. In addition, cancer cells often release HSP90-rich EVs and vesicle-free HSP90, which are potential targets in cancer therapy [19,20,39]. Indeed, HSP90-rich tumor-infiltrating macrophages were detected in oral cancer specimens from patients [20]. The targeted knockdown of HSP90 in oral cancer cells significantly reduced exosome release and transmission efficiency to macrophages [20]. Thus, both cancer cells and TAMs express HSP90 that involves vesicle uptake, phagocytosis, and antigen presentation. Our findings of HSP90α and HSP90β in the macrophage-derived exosomes and as vesicle-free proteins are vital for further studies on the roles of HSP90 in macrophage polarization and the effect of macrophage-derived EVs on cancer cells.

Using the SEC-CF method, we successfully fractionated the various EV fractions. It has been noticed that the precipitating agents may interfere with the structures in EVs, whereas SEC was proved to minimally affect EV composition [28]. In the SEC method, the isolation of vesicles from plasma or serum often caused the co-isolation of high-density lipoprotein (HDL) [40]. Besides, low-density lipoprotein (LDL) can bind onto the isolated EVs. These characteristics often resulted in lipoproteins contaminated in EVs [41]. Consequently, it was difficult to separate the lipoproteins from the EVs using SEC [23,42]. Thus, further development of exosome purification is required, for example, by combining two methods. The combination of the SEC and concentration filters (SEC-CF) is a clue to preparing functional EVs.

Cancer cells are often sensitive to the extracellular microenvironment and signals. We previously showed that cell stress could induce cell morphology changes such as increasing the number of round and spindle-shaped cells, and these morphological changes involve epithelial-to-mesenchymal transition (EMT) [19,38]. A similar EMT-like morphological variation was observed in the present study after treating the oral cancer cells with the fraction 10–20 (containing small exosomes and free proteins such as HSP90). Relevantly, we have shown that prostate cancer-released HSP90-rich EVs initiated EMT in epithelial cells [19]. Heat shock proteins are known as stress proteins. In the current study, macrophage-derived HSP may trigger cell stress on receiver cancer cells leading to morphological change and a reduction in cellular viability. It has been shown that EXO-S, exomeres [15], and other types of proteins generally were contained in the Fr. 10–20 obtained using the SEC method. In addition, EV-based receiver reduction in the ATP content by the CM and EVs might be caused by cargo microRNA, perforin, granzyme, and/or cytokines such as interferon-γ (IFN-γ) [43], tumor necrosis factor α (TNF-α), and effector molecules such as nitric oxide (NO) that are secreted by macrophages. HSPs are molecular chaperones that assist functional protein folding, presumably of anti-tumor factors [38,44,45].

Nonetheless, apart from cytokines, it has been shown that macrophage-derived EVs promote anti-tumor activity. For instance, macrophages are exposed to environmental stresses, such as nutrient deprivation, hypoxia, and the signaling factors, including HSPs, released from cancer cells. These macrophages could recruit diverse intracellular factors and package them into secretory vesicles to mediate the primary response against cancer cells until the immune surveillance system was activated [46]. It was also found that macrophages cultured in glucose-depleted medium secreted human glycyl-tRNA synthetase 1 (GARS1)-EVs, which involved an immunological defense response against tumorigenesis and could promote cancer cell death [46,47]. Relevantly, we emphasize the powerful molecular transferability of macrophage-EVs. The SEC-based EV isolation method shown in the present study indicates the potential roles of macrophage-EVs in targeted therapeutics against tumors.

## 5. Conclusions

In conclusion, the SEC-CF method is useful for purifying exosomes (EXO-S and EXO-L) with high molecular transfer activities, potentially enabling efficient molecular delivery to target cells. In addition, the molecular transfer activities of exosomes from macrophage-like cells can reduce viability in oral carcinoma cells.

## Figures and Tables

**Table 1:** Characters of different sized EV fractions of macrophages, altering recipient cells.

EV/Particle Fraction	Approx. Size	palmGFP Expression	Molecular Transfer ^1^	Approx. Protein Conc.	Marker	ATP Content in Receiver Cells
Fr. 1–3	500–3000 nm	nd.	nd.	0.2 µg/µL	ni.	Decreased
Fr. 4–6	100–500 nm	nd.	Rapid	0.2 µg/µL	ni.	Decreased
Fr. 7–9	80–300 nm	Positive	High	0.2 µg/µL	CD9	Decreased
Fr. 10–12	20–200 nm	nd.	nd.	0.2 µg/µL	ni.	na.
Fr. 13–21	10–70 nm	Positive	High	0.5–2.0 µg/µL	CD63 HSP90α/β	Decreased

^1^ Molecular transfer of palmGFP (approxiomately 30 kD). nd., not detected. ni., not identified. na., not altered.

## Data Availability

The data presented in this study are available in the Supplementary Materials.

## Supplementary Materials

The following are available online at https://www.mdpi.com/article/10.3390/cells10061328/s1: Figure S1: Flow cytometry replication data and analysis of CD14, CD68, CD80, and CD206 in PMA-treated and untreated THP-1 cells. Figure S2: Confocal microscopy of the molecular transfer of palmGFP from THP1/palmG and macrophage/palmG cells to HSC3/palmT in 3D culture. Figure S3: Confocal microscopy of the molecular transfer of palmGFP from macrophage-EVs to receiver macrophages in a 2D culture. Figure S4: Confocal microscopy of molecular transfer of palmGFP from macrophage-EVs to receiver HSC-3 oral carcinoma cells in a 2D culture. Figure S5: Concentration filter method of EV fractions. Figure S6: Full images of the TEM shown in Figure 5A,B. Figure S7: Full images of TEM shown in Figure 5C–E. Figure S8: Full images of Western blot analysis shown in Figure 5H. Figure S9: Full images of Western blot analysis shown in Figure 5I. Figure S10: Particle diameter distribution of macrophage EV fractions used in Figure 5L. Figure S11: Full images of Western blot analysis shown in Figure 8A. Figure S12: Full images of the ArrayScan analysis shown in Figure 8C.
