# Supplementary material for: Exosome-Based Molecular Transfer Activity of Macrophage-Like Cells Involves Viability of Oral Carcinoma Cells: Size Exclusion Chromatography and Concentration Filter Method
Source: Cells. 2021 May 27;10(6):1328. doi: 10.3390/cells10061328 (PMC8228134; doi:10.3390/cells10061328)
Supplement: Supplementary file 1 [file cells-10-01328-s001.zip › cells-1208085-supplementary.pdf]

Supplementary Figures

Exosome-based Molecular Transfer Activity of Macrophage-like Cells Involves Viability of Oral Carcinoma Cells: Size Exclusion Chromatography and Concentration Filter Method

Yanyin Lu [1,2], Takanori Eguchi [1,3,*], Chiharu Sogawa [1,4], Eman A. Taha [1,5,6], Manh Tien Tran [1], Toshiki Nara [7], Penggong Wei [1,8], Shiro Fukuoka [1,9], Takuya Miyawaki [2], Kuniaki Okamoto [1]

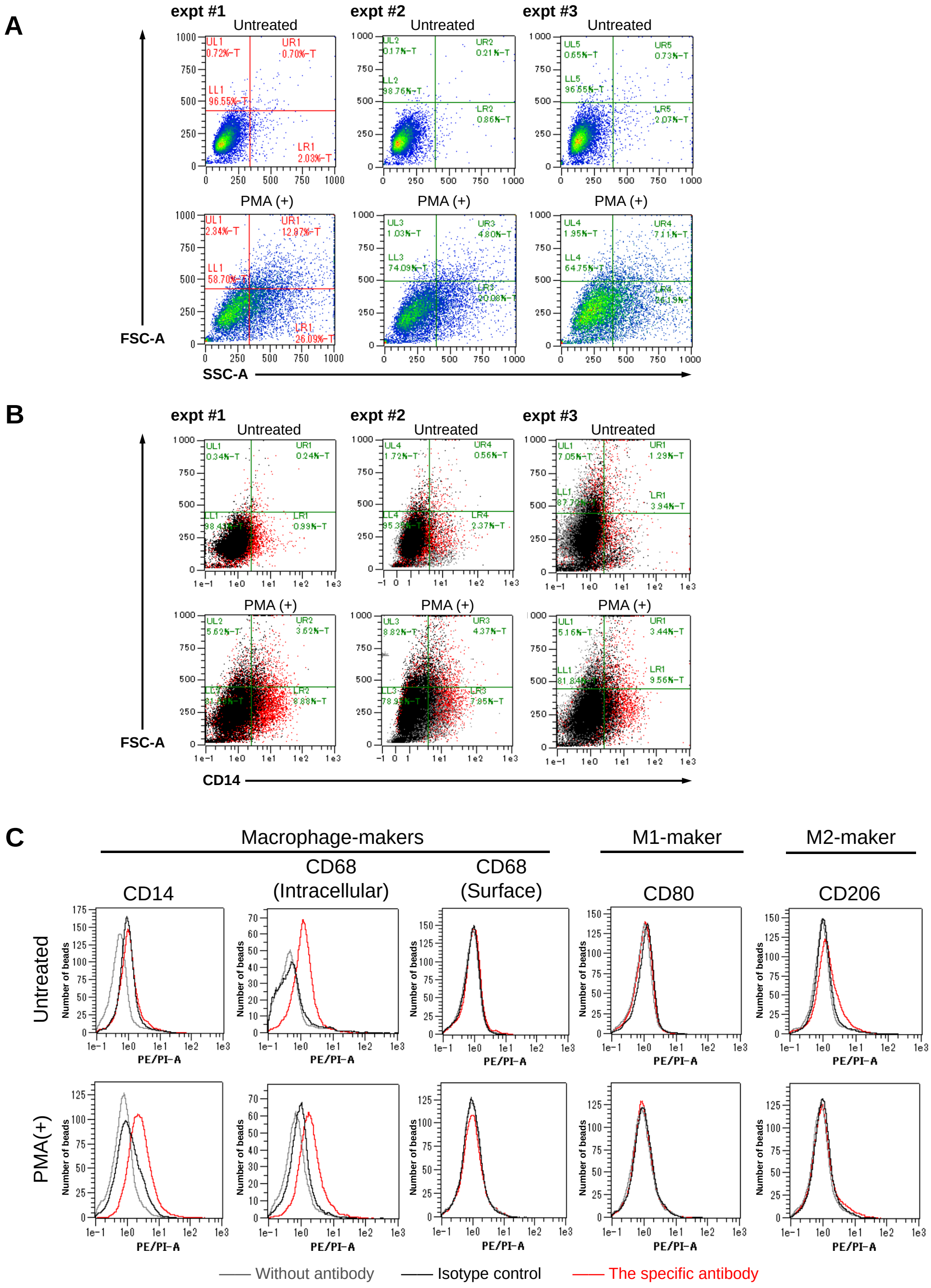

Figure S1: flow cytometry replication data and analysis of CD14, CD68, CD80, and CD206 in PMA-treated and untreated THP-1 cells. (A) Flow cytometry showing size (FSC) and complexity (SSC) of cells. Data were from 3 independent experiments. (B) Flow cytometry detecting cell surface CD14. Data are representative of three independent experiments. (C) Flow cytometry for M0, M1, and M2 type macrophages.

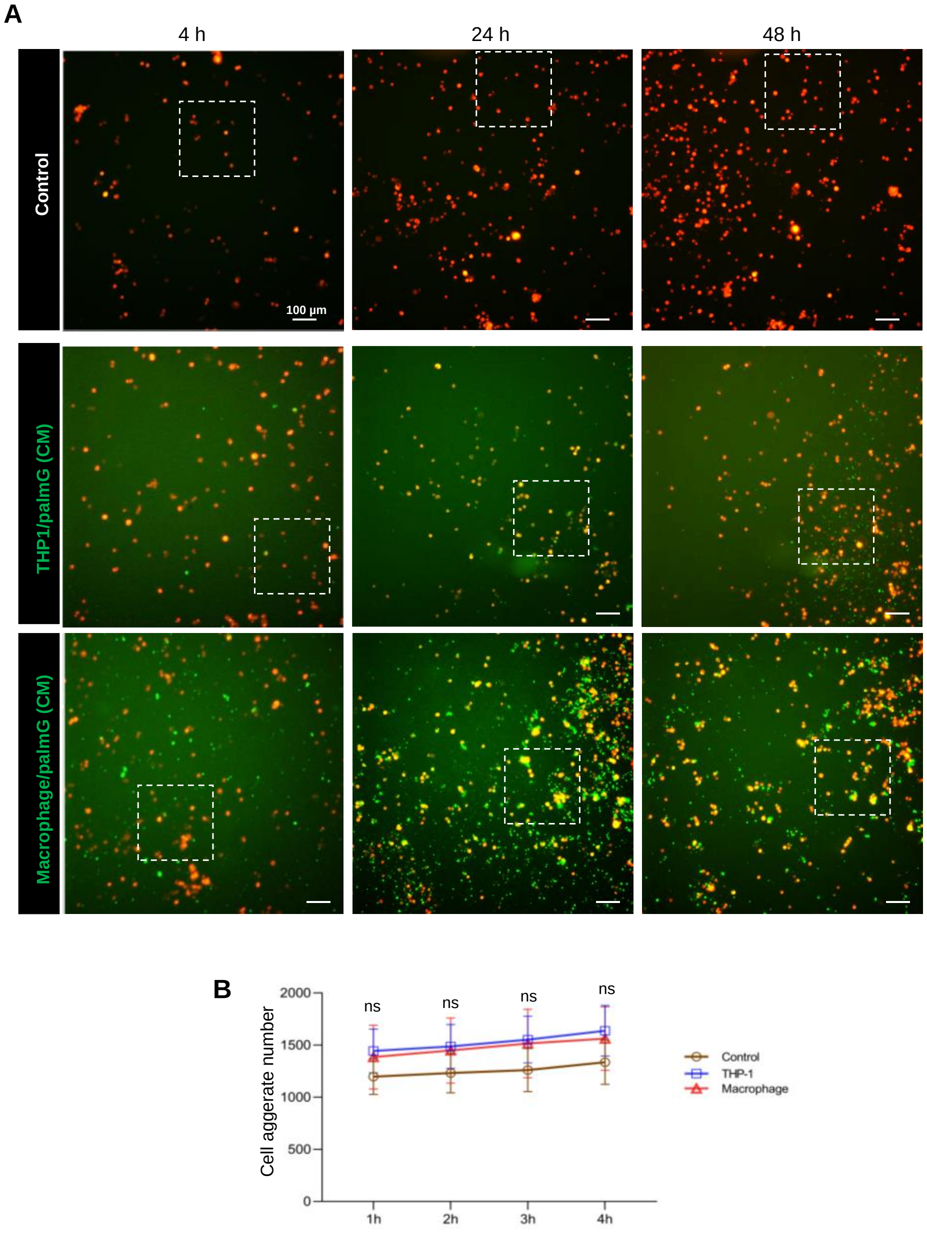

Figure S2: confocal microscopy of molecular transfer of palmGFP from THP1/palmG and macrophage/palmG cells to HSC3/palmT in 3D culture. (A) Full images of ArrayScan supporting Figure 2C. Dashed border: the field shown in Figure 2C. (B) The number of cell aggerates within the first 4 h supporting Figure 2C. No significant difference was found between the numbers of cell aggregates of each group.

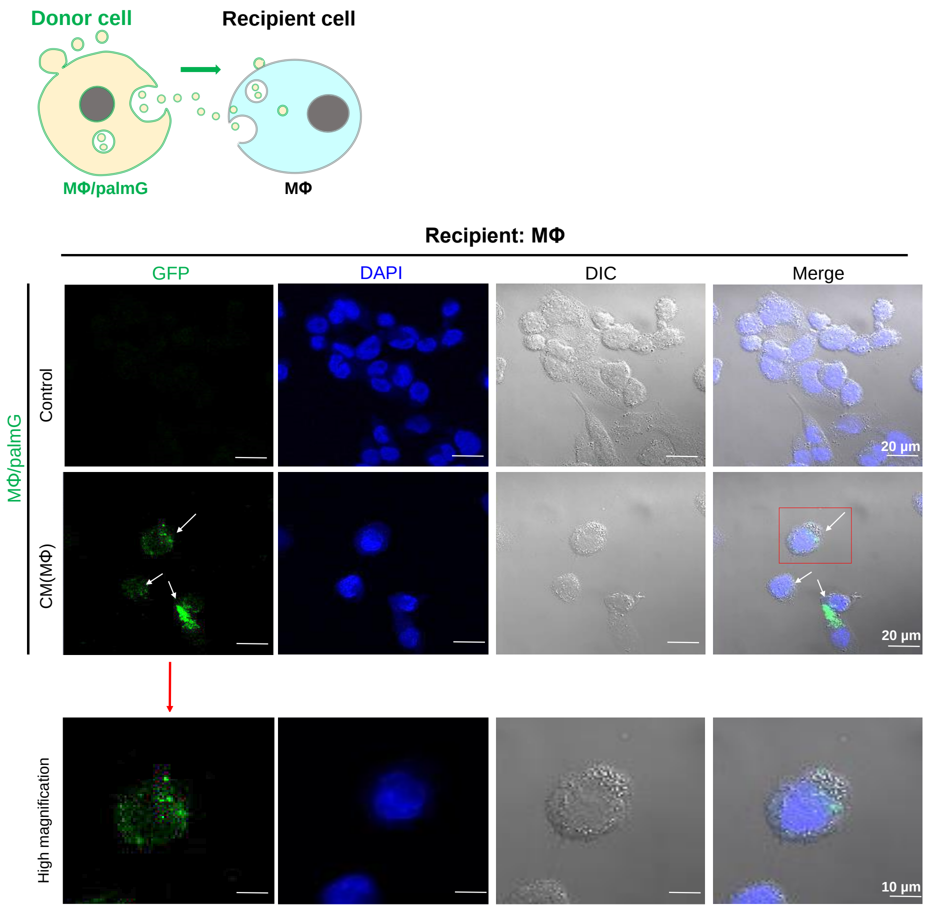

Figure S3: confocal microscopy of the molecular transfer of palmGFP from macrophage-EVs to receiver macrophages in 2D culture. Arrow, palmGFP transferred.

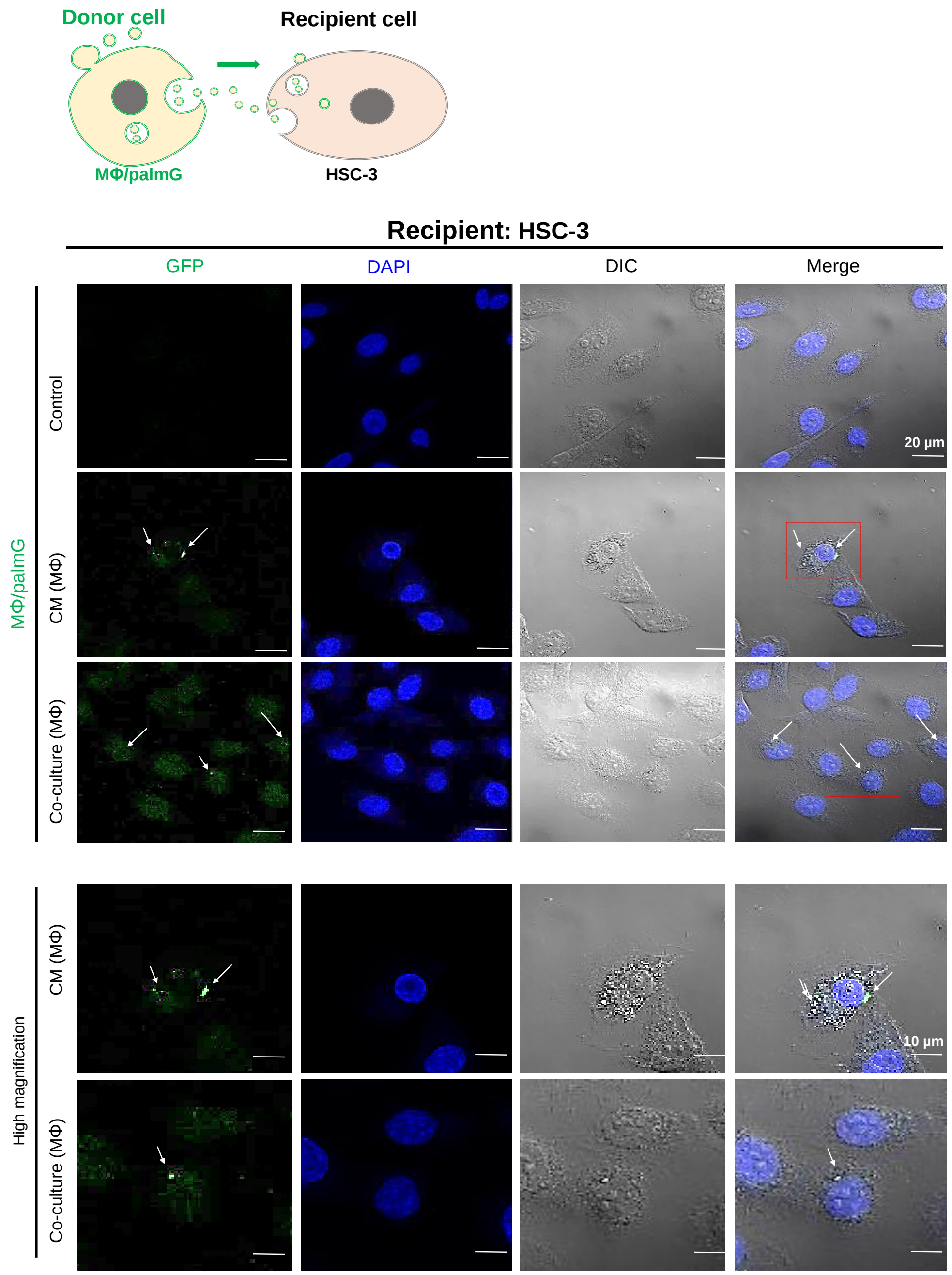

Figure S4: confocal microscopy of the molecular transfer of palmGFP from macrophage-EVs to receiver HSC-3 oral carcinoma cells in 2D culture. Arrow, palmGFP transferred.

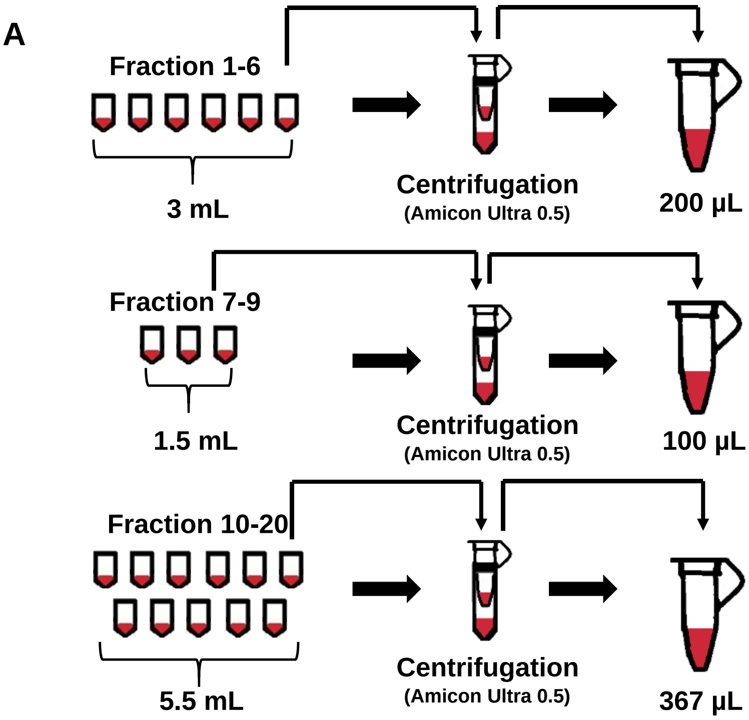

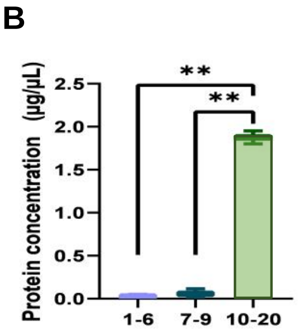

C

Protein concentration of various EV fractions			
Samples	Conc. (µg/µL)	Vol (µL)	Total protein applied to WB (µg)
Fr. 1—6	0.05	30	1.5
Fr. 7—9	0.12	30	3.5
Fr. 10—20	2.0	30	58.5
PBP-EVs	4.0	7.6	30.1
non-EV	1.0	30	30.5

Figure S5: Concentration filter method for EV fractions. (A) Fr. 1-6 were gathered into a single tube and then concentrated using ultrafiltration. Fr. 7-9 and Fr.10-20 were also concentrated by the same method. (B) Protein concentrations in the fraction 1-6, 7-9, and 10-20. N=2. **P<0.01. (C) A table showing the protein concentrations of the fractions and protein amount applied in western blotting.

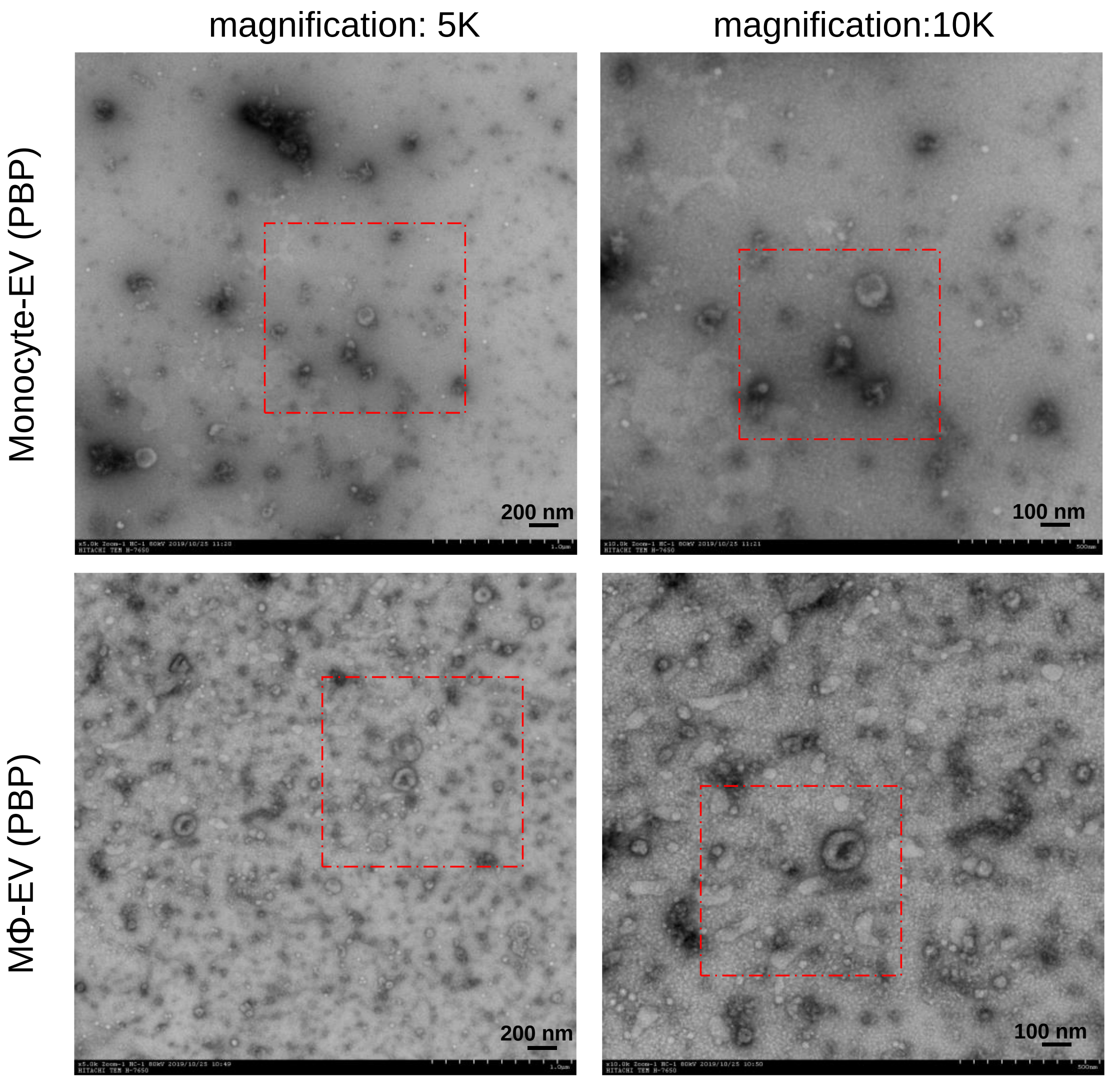

Figure S6: full images of TEM shown in Figure 5A and 5B. Dashed border: the field shown in Figure 5A and 5B.

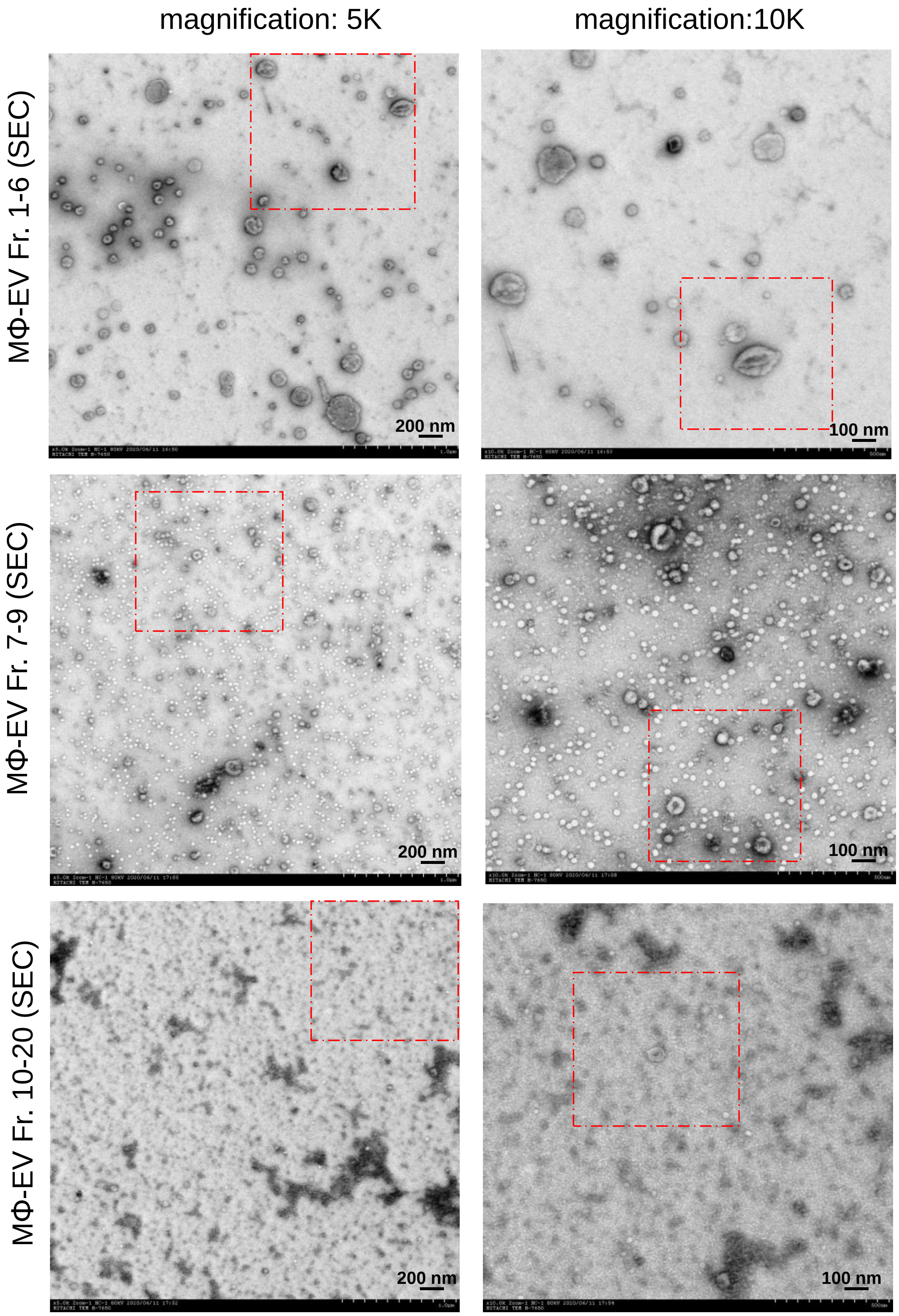

Figure S7: full images of TEM shown in Figure 5C, 5D and 5E. Dashed border: the field shown in Figure 5C, 5D and 5E.

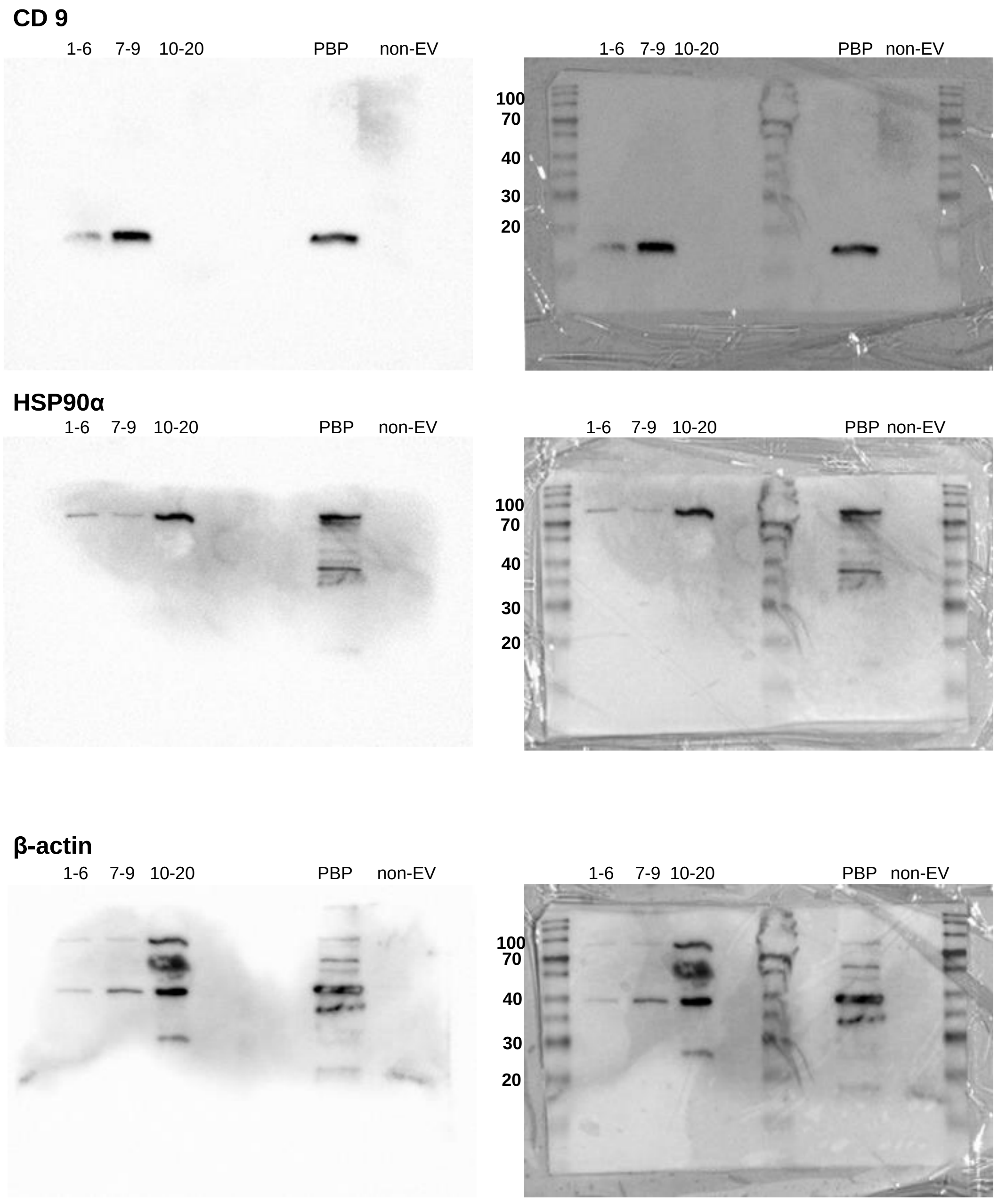

Figure S8: full images of western blot analysis shown in Figure 5H

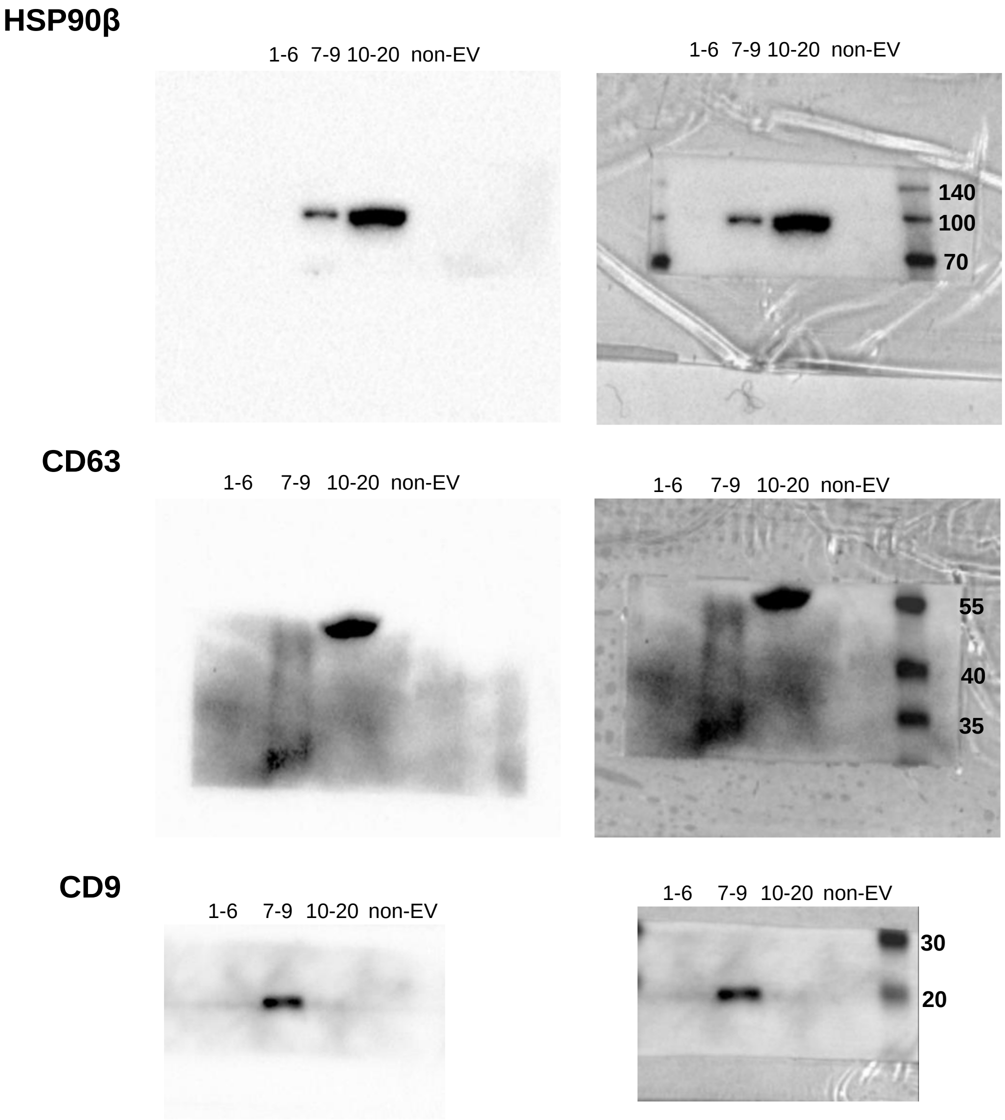

Figure S9: full images of western blot analysis shown in Figure 5I.

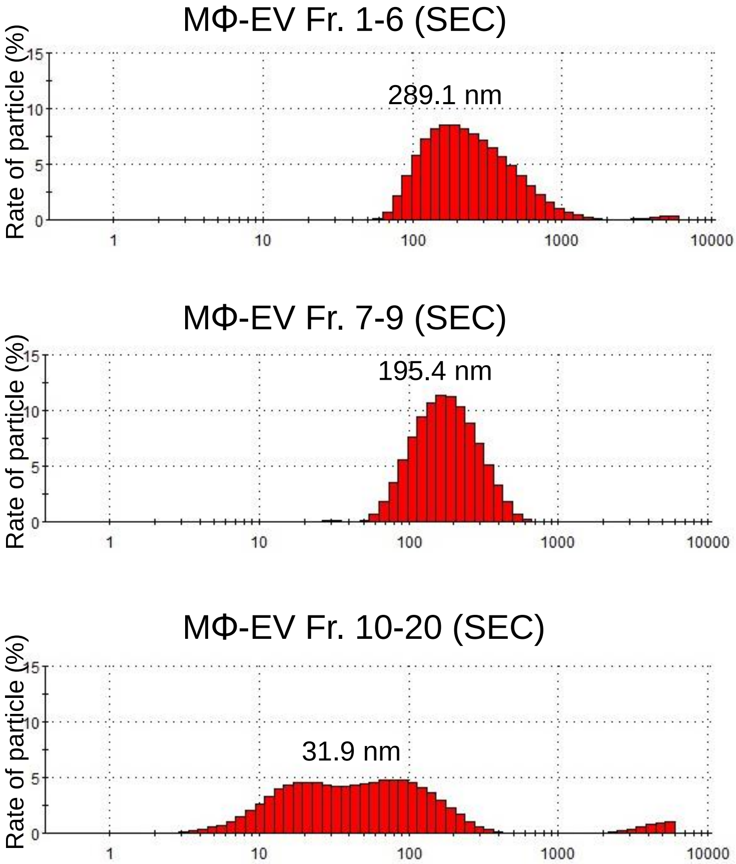

Figure S10: particle diameter distribution of macrophage EV fractions used in Fig 5L.

Protein concentration of various EV fractions from macrophage/PalmG			
Samples	Conc. (μg/μL)	Vol (μL)	Total Protein (μg)
1-3	0.13	20	2.7
4-6	0.19	20	3.7
7-9	0.21	20	4.2
10-12	0.18	20	3.6
13-15	0.66	20	13.3
16-18	1.3	20	26.0
19-21	0.82	20	16.4
1-21	5.3	5	26.6

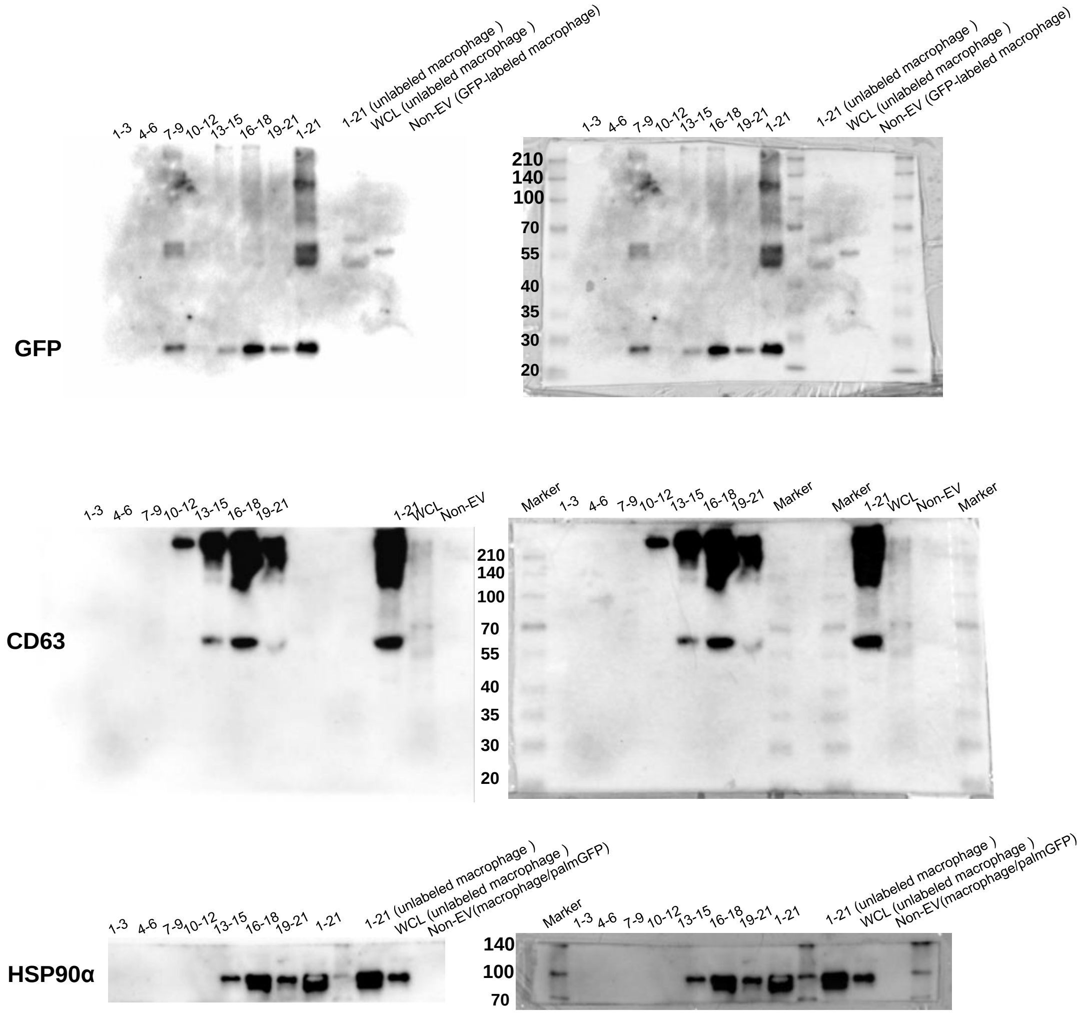

Figure S11: full images of western blot analysis shown in Figure 8A.

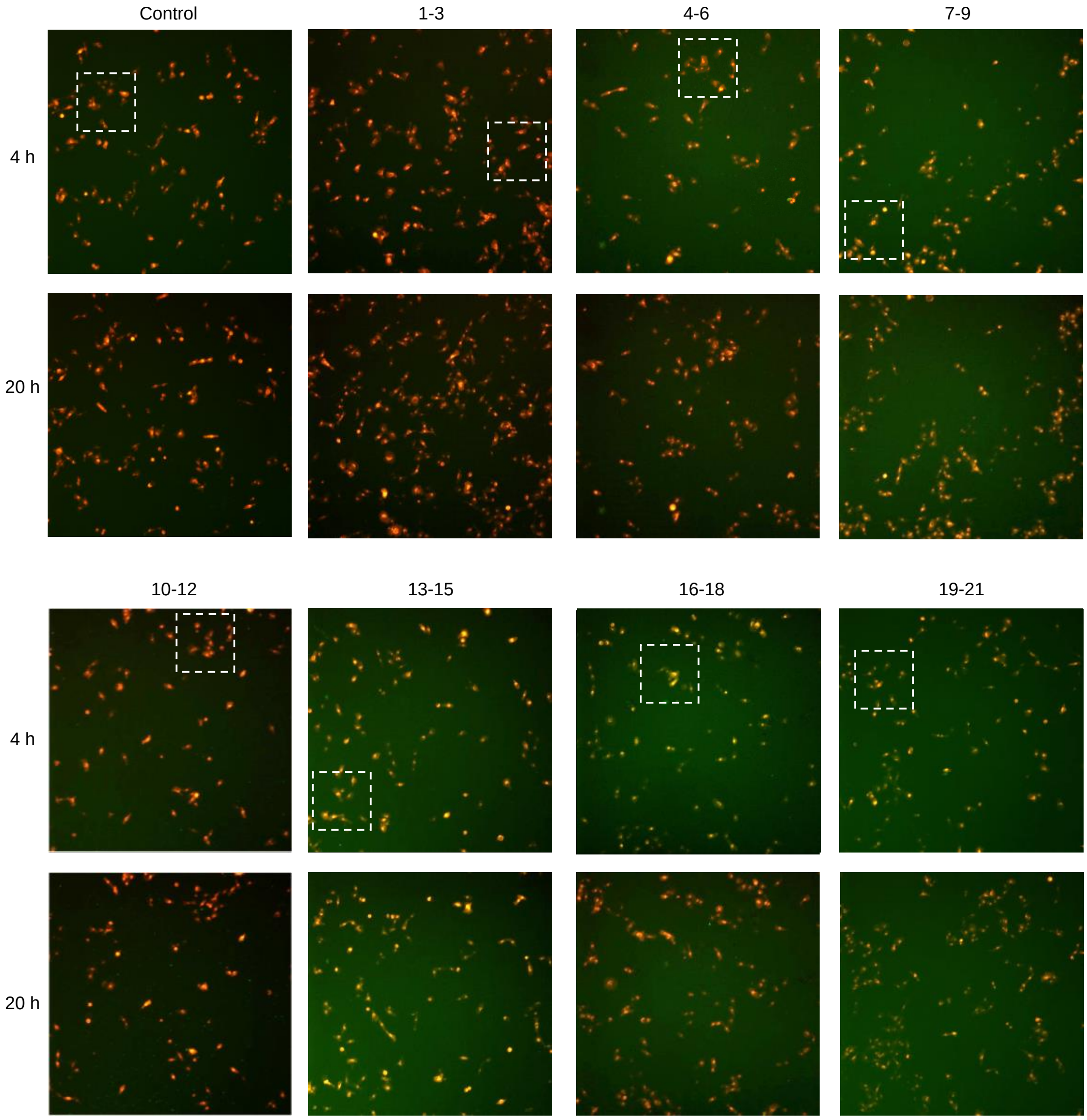

Figure S12: Full images of representative field data obtained in ArrayScan shown in Figure 8C. Dashed border: the field shown in Figure 8C.
